# Characterising Epigenetic Tipping Points using a Spectral Dimension Reduction Approach

**DOI:** 10.1007/s11538-026-01602-w

**Published:** 2026-03-18

**Authors:** Tomás Alarcón, Javier A Menendez, Josep Sardanyés

**Affiliations:** 1 Institució Catalana de Recerca i Estudis Avançats (ICREA), Barcelona, 08012 Spain; 2 Centre de Recerca Matemàtica, Bellaterra, 08193 Spain; 3 Departament de Matemàtiques, Universitat Autonòma de Barcelona, Bellaterra, 08193 Spain; 4 Barcelona Collaboratorium for Theoretical & Predicitive Biology, Barcelona, 08005 Spain; 5 Program Against Cancer Therapeutic Resistance (ProCURE), Girona, 17007 Spain; 6 Girona Biomedical Research Institute, Salt, 17190 Spain

**Keywords:** Epigenetic regulation, Spectral dimension reduction, Tipping points

## Abstract

Epigenetic landscapes (ELs) are defined by the pattern of epigenetic marks (acetylation, methylation, etc.) layed over large chromatin regions. The information contained in the ELs is essential to sustain the patterns of gene expression that shape cell fate and identity. EL maintenance requires the precise regulation of chromatin-modifying enzymes (ChME) and their metabolic cofactors (McF). Competition for ChME or dysregulation of McF abundance can lead to degradation of ELs, triggering large-scale changes in the cell fate information contained in EL. Thus, predicting impending epigenetic tipping points (ETPs) by identifying early warning signals (EWS) may help to anticipate the onset of cell identity loss during aging and cancer. Since ELs are formed (and maintained) by a systems of writer/eraser enzymes that interact both in *cis* (local) and *trans* (long-range) modes, their mathematical description involves a high-dimensional dynamical system, where identifying ETPs and characterising the biological mechanisms that control them remains challenging. Here, we develop a general mathematical framework that incorporates different connectivity patterns generated by the 3D chromatin folding structure to analyze competition-induced ETP in large EL. This framework allows us to measure the sensitivity and robustness of ETP to the availability of metabolic cofactors and to identify potential EWS. Using a dimension reduction method, we derived coarse-grained (CG) equations for the collective observables associated with chromatin modifications. Analysis of the CG system allows the prediction of global transitions that shape the large-scale features of EL, accurately reproduce the corresponding microscopic benchmarks, and reveal the existence of tipping points under conditions of ChME competition. We applied the CG method to predict ETP under different connectivity patterns, including heterogeneous profiles such as those found in Hi-C data. Although a robustness measure for stable EL was derived from the CG dynamics in bistable regimes, sensitivity analysis revealed that metabolic cofactors have the greatest impact on EL robustness. In particular, we identified the metabolic cofactors SAM and acetyl-CoA as potential EWS for the catastrophic loss of hyperacetylated EL induced by ChME competition. The ability to predict global ETP can facilitate the discovery of predictive biomarkers and inform metabolic interventions aimed at limiting and reversing pathological cell fate decisions.

**Supplementary Information:**

The online version contains supplementary material available at 10.1007/s11538-026-01602-w.

## Introduction

Multicellular organisms are composed of many different cell types that perform specialized functions and are characterized by specific patterns of gene expression. Due to intrinsic stochasticity and environmental cues, heterogeneity can compromise gene expression patterns associated with specific cell identities. To prevent a cellular identity crisis, cells have evolved several regulatory mechanisms that tightly control gene expression, involving transcriptional, translational, post-translational, and epigenetic elements. In particular, maintaining stable epigenetic landscapes (EL) is essential to confer specific fates and functions to cells and requires the timely regulation of chromatin modifiers, including epigenetic enzymes and their associated metabolic cofactors. Dysregulation of chromatin-modifying enzymes and metabolic fluxes could drive the epigenetic regulatory system through ”tipping points” that induce sudden and large transitions in the EL. Such critical transitions can fundamentally alter both the structure and the information contained within the EL, thereby leading to changes in cellular identity. The deterioration of the information storage in (and retrieval from) the EL may drive physiological responses such as age-related tissue dysfunction (Magalhaes 2023) or key phenotypic switches such as the epithelial-to-mesenchymal transition (EMT), which plays important physiological roles in embryonic development, tissue regeneration, and wound healing, but also occurs in pathological processes such as cancer progression and organ fibrosis (Al-Radhawi et al. 2022).

Global EL transitions can be induced in response to variations in chromatin modifiers (Butz et al. 2022) or changes in chromatin structure due to dynamic rewiring of the interaction map between genome regions (Joshi et al. 2015). Competition for chromatin modifiers has been identified as a candidate for inducing large-scale global transitions, where large changes in ELs occur in response to small changes in a given regulatory state (Stepanova et al. 2025a). According to the ”relocalization of chromatin modifiers hypothesis”, the accumulation of DNA damage may lead to excessive competition for dual-function epigenetic enzymes such as sirtuins, which facilitate DNA repair at DNA double-strand breaks (DSBs), but also help to epigenetically maintain specific patterns of gene expression, particularly at developmental genes (Yang et al. 2023). The establishment of a positive feedback loop of chromatin modifications in response to DNA damage has been proposed to promote the erosion of ELs and accelerate the loss of cellular identity that drives aging (Stepanova et al. 2025b). Another example of global transitions in ELs governed by competition between histone modifications and transcription factor dysregulation is the EMT phenotypic switch in which epithelial cells lose cell-cell adhesion and apico-basal polarity to gain migration and invasion properties (Al-Radhawi et al. 2022). Such qualitative transitions in the ELs may indeed represent tipping points, i.e., phase transitions that alter the global structure of an EL.

Tipping points (TPs) refer to abrupt and often irreversible shifts in the state of a system that occur once a critical threshold is crossed (Sardanyés et al. 2024). Although the concept of TPs is broadly defined in the scientific literature, two closely related interpretations are most commonly used. The first interpretation characterizes TPs as catastrophic bifurcations in a mathematical sense, often referred to as bifurcation-induced tipping (B-tipping). Under this view, a tipping point corresponds to a critical value of a control parameter beyond which the system abruptly transitions to an alternative state, typically one that is monostable. Numerous ecological models demonstrate that such catastrophic shifts are most frequently associated with saddle-node bifurcations, which are local bifurcations. Although saddle-node bifurcations represent the most common mechanism underlying abrupt transitions between alternative states, other types of bifurcations, including global bifurcations, can also lead to catastrophic changes. The second interpretation defines tipping points as unstable equilibrium states, often conceptualized as peaks in a potential landscape. In this framework, the transition between states does not necessarily require a parameter to cross a critical value; instead, it may be triggered by external disturbances or stochastic fluctuations. Such transitions are commonly referred to as noise-induced tipping (N-tipping), where perturbations push the system away from an unstable equilibrium toward an alternative stable state. Within the context of this analysis, we will focus on B-tipping, specifically, on TPs induced by saddle-node bifurcations.

All of the above evidence highlights the emerging recognition that competition between multiple sources of epigenetic variability (such as chromatin structure, histone modifications, transcription factor binding, etc.) is a key driver of EL transitions and maintenance versus loss of epigenetic information. However, we lack a general theoretical framework to analyze the behavior of (competition-induced) tipping points in large-scale ELs, to identify the sensitivity and the robustness of such ELs to epigenetic variability such as the fluctuations in metabolic cofactors, and to potentially identify early warning signals (EWS). EWS, which typically arise when a nonlinear system approaches a tipping point, involve changes in the dynamics associated with variability, memory, return times, and flickering (Dakos et al. 2012; Carpenter and Brock 2006). Although EWS have been extensively studied in complex ecological and global climate systems (Lenton et al. 2008; Scheffer et al. 2009, 2012; Cline et al. 2014; Ditlevsen and Ditlevsen 2023; Sardanyés et al. 2024), their use in predicting the onset of disease is only beginning to be explored as evidence accumulates that in complex diseases such as cancer, autoimmunity, cystic fibrosis, AIDS, influenza, and others, deterioration is typically abrupt rather than smooth (Liua et al. 2014; Liu et al. 2017; Chen et al. 2012). Like ecosystems, molecular biosystems exhibit qualitative transitions in their response to stochastic perturbations but show little sign of the impending switch from a healthy to a diseased state until it has occurred. Therefore, to predict these critical transitions, it is crucial to identify EWS capable of capturing the proximity to tipping points.

**Fig. 1:**
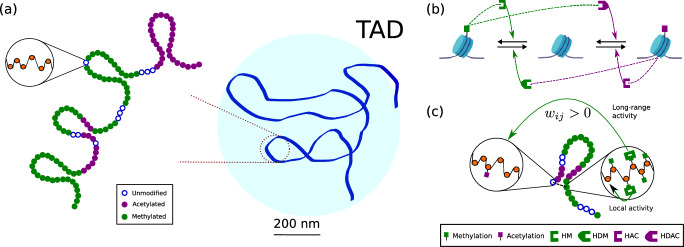
**Schematic representation of the microscopic model of chromatin modifications with local and long-range activity.** (a) DNA is organized in a complex, multiscale fashion, from chromosomes to sequence. Here, we focus on the scale of topologically-associating domains (TADs), which are regions with higher-than-average inner connectivity and which are loosely connected with genomic regions outside the TAD. TADs exhibit a rather large degree of variation in sizes, ranging between 100-5000 kb (Zufferey et al. 2018; Szabo et al. 2019). Based on quantitative information for Hi-C maps, the resolution of such maps is of the order of 1 kilobase (kb) (Liu et al. 2021). Since each nucleosome is wrapped with DNA of approximately 150 bases in length, each region in our model contains between 5 and 10 nucleosomes. Since each nucleosome contains two H3 histones, this implies that the number of H3K27 sites available for modification, $S_0$, is of the order of 10-20. (b) Negative and positive feedback on chromatin modifiers by epigenetic modifications play a crucial role in generating and maintaining stable epigenetic landscapes. The feedbacks that we are incorporating in our model are shown in panel (c) (adapted from Dodd Dodd et al. (2007), see also (Al-Radhawi et al. 2022)). As illustrated in (c), such feedbacks can be either *cis* (local, within a region) or *trans* (non-local, between regions). We assume that the network of physical contacts between regions (represented here by the dashed line) provides the backbone for such feedbacks.

Patterns of chromatin modifications have been studied using theoretical models in small genomic regions on the order of a few kilobases (Newar et al. 2022), and, less frequently, in larger systems (Sneppen and Ringrose 2019). These models are computational in nature, and their analysis for stability or parameter sensitivity can only be performed by extensive (and often costly) numerical simulation. As a first step to remedy this situation, we address here the development of a framework that allows for analytical and feasible computational approaches. Using dimension reduction techniques, we derive coarse-grained (CG) evolution equations for suitably defined collective observables associated with specific chromatin modifications. By analyzing the CG model, both in the stochastic and mean-field regimes, our framework can predict abrupt, global transitions in the large-scale features of ELs (see Fig. 1).

In particular, we show that the predictions of the CG model robustly and accurately reproduce those of the corresponding microscopic benchmark. Specifically, we demonstrate the existence of tipping points under conditions of competition for chromatin modifiers. In multi-stable regimes, the asymptotic analysis of the (stochastic) low-dimensional, coarse-grained dynamics uniquely allows us to derive a novel robustness measure for stable ELs. Our framework integrates several biological variables including competition for chromatin modifiers, metabolic cofactors, and the connectivity patterns generated by the 3D folding structure of chromatin on the robustness of the EL. Because our framework can be treated analytically, we can gain a deeper understanding of how these three components interact not only in the generation of robust EL, but also in EL erosion leading to loss of epigenetic information. By performing a sensitivity analysis of the robustness measure, we extend our analysis to determine the model parameters with the greatest impact on the loss of robustness for specific ELs. Such an analysis allows us to identify that the availability of two metabolic cofactors for chromatin-modifying reactions, SAM and Acetyl-CoA, strongly influences the robustness of specific ELs and thus correlates closely with EWS for predicting the proximity of EL tipping points. Our methodology may help to guide metabolic interventions to prevent or even reverse undesired global EL transitions that underlie the collapses of cellular identity in aging and cancer.

## Materials & Methods

### Microscopic Mathematical Model of Chromatin Modifications

We start our description of materials and methods by introducing the so-called *microscopic* model. We consider a simplified model that solely takes into account modifications of H3K27, specifically acetylation and methylation (Dodd et al. 2007; Newar et al. 2022). In this model, we consider a chromatin region divided into a number of sub-regions, as shown in Fig. 1(a). Each of these regions contains a number of nucleosomes. Since each nucleosome contains two H3 histones the number of sites available for modification within each subregion, $S_{27}$, is twice the number of nucleosomes. Typical figures for these quantities are given in Fig. 1. The microscopic model keeps track of the stochastic dynamics of the number of modified sites within each sub-region.

Models based on principles and premises similar to the one presented here can be found in the literature (see e.g. Al-Radhawi et al. 2022; Stepanova et al. 2025a, b; Newar et al. 2022; Sneppen and Ringrose 2019; Dodd et al. 2007; Folguera-Blasco et al. 2018, 2019; Alarcón et al. 2021; Sneppen et al. 2008; Sneppen and Dodd 2012, 2016), specifically regarding enzyme kinetics and the presence of rate-limiting metabolic cofactors. Many of these models exhibit transitions of the type studied in this paper (see Al-Radhawi et al. 2022; Stepanova et al. 2025a, b). We note that the main aim of the current study is to provide a general framework that allows us to study in detail the existence and features of such transitions by developing a stochastic version of the spectral dimension reduction method. To illustrate the use and usefulness of this method for large-scale transitions in the epigenetic-regulatory states of large chromatin regions, we use the specific model we discuss in this section.

We formulate a stochastic dynamical system for the number of acetylated and methylated residues within a specific region. We number such regions according to $i=1,\dots ,N_C$, where $N_C$ is the total number of regions considered in our simulations (see Fig. 1(a)). Chromatin modifications involve the addition and removal of covalent modifications and, therefore, are mediated by enzymes. Thus, at each site $i=1,\dots ,N_C$, we consider the Michaelis-Menten (MM) model of enzyme kinetics to model each of the chromatin modifications (i.e., addition/removal of methylation, addition/removal of acetylation) (Folguera-Blasco et al. 2018, 2019; Alarcón et al. 2021; Al-Radhawi et al. 2022). The MM model assumes that the substrate and the enzyme form a complex that then relaxes into a product (modified chromatin) and returns the enzyme. Specifically, the addition of (tri)methylation and acetylation marks to H3K27 is modeled by:respectively. In this case, the substrates, $\textbf{S}=\{S_i\}_{i=1}^{N_C}$, are unmodified H3K27 residues. Methylation is mediated by EZH2, the enzymatically active member of the polycomb repressor complex, $E_2$, while its acetylation is regulated by a histone acetyl transferase (HAT), $E_H$. The complexes $\mathbf{C_E}=\{C_{E_i}\}_{i=1}^{N_C}$ and $\mathbf{C_H}=\{C_{H_i}\}_{i=1}^{N_C}$, which quantify the amount of chromatin-bound enzyme, relax onto $\textbf{M}=\{M_i\}_{i=1}^{N_C}$ and $\textbf{A}=\{A_i\}_{i=1}^{N_C}$, which refer to the methylation and acetylation patterns within the chromatin region of interest, respectively.

Demethylation and deacetylation are modeled using the same principles:i.e., histone demethylase UTX, $E_U$ bind to their substrates, $M_i$, to form the complexes $\mathbf{C_U}=\{C_{U_i}\}_{i=1}^{N_C}$ and relax onto their unmodified form, $S_i$. The same process involving histone deacetylase (NURD or members of the SIRT family), $E_S$, and substrate $A_i$ leads to the removal of acetylation marks via formation of the complexes $\mathbf{C_S}=\{C_{S_i}\}_{i=1}^{N_C}$.

Furthermore, as it has been described in previous studies (Dodd et al. 2007; Sneppen et al. 2008; Sneppen and Dodd 2012, 2016; Sneppen and Ringrose 2019) the rate at which modifications occur in region *i* is regulated by the landscape of epigenetic marks, both locally (*cis*) and non-locally (*trans*) (see Fig. 1(b) and (c)) (Szabo et al. 2019; Newar et al. 2022). We consider that the map of contacts provided by Hi-C datasets also provides a physical backbone for the biochemical interactions. In other words, we will assume that the modification rates at region *i* are regulated by the marks in *j* if $w_{ij}>0$, i.e., if both regions are in physical contact. The quantities $w_{ij}$ are the components of the matrix $\textsf{W}=(w_{ij})$ and quantify the frequency with which regions *i* and *j* are in contact. We assume that such feedbacks affect the ability of chromatin modifications to facilitate or hinder the binding of the modifiers to their substrates. In general, feedbacks are such that acetylation (methylation) marks increase the rate at which further acetylation (methylation) occurs and inhibit the rate of addition of methylation (acetylation) marks. Specifically, feedbacks are incorporated in our model through the rates of binding and unbinding of the epigenetic enzymes to their substrates:

- Binding of EZH2 to unmodified H3K27 is favored by methylated residues within the neighbours of *i*:
  1 $$\begin{aligned} k_{1_i}=k_1\left( \alpha _1+\sum _jw_{ij}M_j\right) \end{aligned}$$
- Binding of UTX to methylated H3K27 is favoured by acetylated residues within the neighbours of *i*:
  2 $$\begin{aligned} k_{4_i}=k_4\left( \alpha _4+\sum _jw_{ij}A_j\right) \end{aligned}$$
- Binding of HAC to unmodified H3K27 is favoured by acetylated residues within the neighbours of *i*:
  3 $$\begin{aligned} k_{7_i}=k_7\left( \alpha _7+\sum _jw_{ij}A_j\right) \end{aligned}$$
- Binding of SIRT to unmodified H3K27 is favoured by methylated residues within the neighbours of *i*:
  4 $$\begin{aligned} k_{10_i}=k_{10}\left( \alpha _{10}+\sum _jw_{ij}M_j\right) \end{aligned}$$

The matrix of connectivity $\textsf{W}$ plays a central role in the model. It reflects the 3D folding structure of chromatin and provides a scaffold for long-range propagation of epigenetic modifications (Jost and Vaillant 2018). To illustrate the breadth of applicability of our method and also the effects of considering different structures, we will consider three patterns of connectivity, namely, all-to-all, first-neighbors, and heterogeneous. The all-to-all connectivity corresponds to a pattern of interactions where two sites are, on average, equally likely to be connected regardless of the distance between them. The intensity of the interaction is also (on average) distance-independent. First-neighbors interactions refer to the case where neighbor sites (along the chromatin chain) have strong interactions, whereas sites beyond first neighbors are very weakly connected. Heterogeneous connectivity is associated with cases where some subregions are very tightly connected, whereas between-subregion interactions are very weak. Hi-C maps belong to the heterogeneous class (Szabo et al. 2019; Lawson et al. 2023). For the current discussion, we will consider that $\textsf{W}$ is a constant input to the model and analyze the effect of considering different architectures. Details on how we generate the $\textsf{W}$ matrices are provided in Supplementary Materials, Section II.

Finally, we consider the problem of large-scale transitions (tipping points) induced by competition for chromatin modifiers in ELs. Such situations have been studied, for example, in the context of aging (Yang et al. 2023), where sirtuins (that have HDAC activity) are recruited to DNA-damaged sites and the epithelial-to-mesenchymal transition (Al-Radhawi et al. 2022). For concreteness, we will focus on the case in which there is competition by sirtuins. We consider a simple, binding-unbinding model of sequestration of SIRT at double-strand breaks (DSBs), where a fixed amount of DSBs, *D*, captures SIRT, i.e.,where $C_{S_D}$ is the amount of SIRT captured by DSBs. For simplicity, we will assume that this reaction is very fast and we can consider that $C_{S_D}$ is in quasi-equilibrium.

The stochastic dynamics of the microscopic model tracks the evolution of the state of the system at time *t* is determined by the value of $\textsf{X}(t)=(X_{ij})$:

$$\begin{aligned} \textsf{X}=\left( \textbf{M}^T,\textbf{A}^T,\textbf{S}^T,\mathbf{E_2}^T,\mathbf{C_E}^T,\mathbf{E_U}^T,\mathbf{C_U}^T,\mathbf{E_H}^T,\mathbf{C_H}^T,\mathbf{E_S}^T,\mathbf{C_S}^T,\mathbf{C_{S_D}}^T\right) ^T, \end{aligned}$$

with $i=1,\dots ,N_S$ and $j=1,\dots ,N_C$ ($N_C$ is the number of regions within the chromosome being considered, and $N_S=12$).

The stochastic dynamics is given by Gillespie (2000); Anderson and Kurtz (2010):

$$\begin{aligned} X_{ij}(t)=X_{ij}(0)+\sum _{k=1}^Rr_{ijk}Y\left( \int _0^tW_{(j-1)R+k}(\textsf{X})ds\right) \end{aligned}$$

where $W_k$ and $\textsf{r}_k$ are defined as:

$$\begin{aligned} P\left( \textsf{X}(t+\Delta t)=\textsf{X}(t)+\textsf{r}_k\vert \textsf{X}(t)\right) =W_k(\textsf{X}(t))\Delta t+{\mathcal O}(\Delta t^2), \end{aligned}$$

and $Y(\lambda )\sim \text{ Poisson }(\lambda )$. The rates, $W_k$, and the corresponding stoichiometric coefficients, $\textsf{r}_k$, are given in Table 2.

To carry on with our analysis, we first consider that our system exhibits separation of (time) scales (see Appendix A). As we have shown in detail elsewhere (Alarcón 2014; Folguera-Blasco et al. 2018, 2019; Alarcón et al. 2021), under the scaling assumptions specified in Appendix A, the stochastic evolution of the system can be rewritten as:

5 $$\begin{aligned} &  x_{ij}(\hat{t})=x_{ij}(0)+\sum _{k=1}^Rr_{ijk}\frac{1}{\Omega _{27}}Y\left( \Omega _{27}\int _0^{\hat{t}}\omega _{(j-1)R+k}(\textsf{x})ds\right) ,\;i=1,2,3 \end{aligned}$$

6 $$\begin{aligned} &  x_{ij}(\hat{t})=x_{ij}(0)+\sum _{k=1}^Rr_{ijk}\frac{1}{\Omega _{E}}Y\left( \frac{1}{\epsilon }\Omega _{E}\int _0^{\hat{t}}\omega _{(j-1)R+k}(\textsf{x})ds\right) ,\;i=4,\dots ,N_S \end{aligned}$$

where $$\hat{t}=k_2\Omega _E\Omega _{27}t$$, and

$$\begin{aligned} x_{ij}=\left\{ \begin{array}{l}\Omega _{27}^{-1}X_{ij} \text{ if } i=1,2,3\\ \Omega _{E}^{-1}X_{ij} \text{ if } i=4,\dots ,N_S\end{array}\right. \end{aligned}$$

The small parameter $\varepsilon$ is $\varepsilon = \Omega _E/\Omega _{27} \ll 1$. The specific dependence on $\Omega _E$ and $\Omega _{27}$ is such that the mean-field limit (which, in this case, corresponds to taking $\Omega _E\rightarrow \infty$ and $\Omega _27\rightarrow \infty$ with $\varepsilon =$cnt.$\ll 1$) is well defined. The mean-field limit for Markov-jump processes (MJPs) is predicated upon the law of large numbers for the Poisson distribution. Let $Y(\hat{\lambda })$ be a Poisson random number whose PDF with parameter equal to $\hat{\lambda }$. Let us assume that $\hat{\lambda }$ scales with system size, $\Omega$, as $hat{\lambda }~\Omega \lambda$. Then, as $\Omega \rightarrow \infty$:

$$\begin{aligned} \Omega ^{-1}Y(\Omega \lambda )\rightarrow \lambda \end{aligned}$$

(see Anderson and Kurtz 2010 for details). Using this property, one can take the mean-field limit in a rigorous way, which is why $\Omega _27$ and $\Omega _E$ must remain in the equations. This scaling, more details of which are given in Appendix A, is standard in MJPs in general (Anderson and Kurtz 2010) and also in MJPs with multiples time scales (Kang et al. 2014).

For simplicity, in what follows we remove the ”hat” notation, so that $\hat{t}\rightarrow t$.

### Dimensional Reduction of the Microscopic Model

**Fig. 2:**
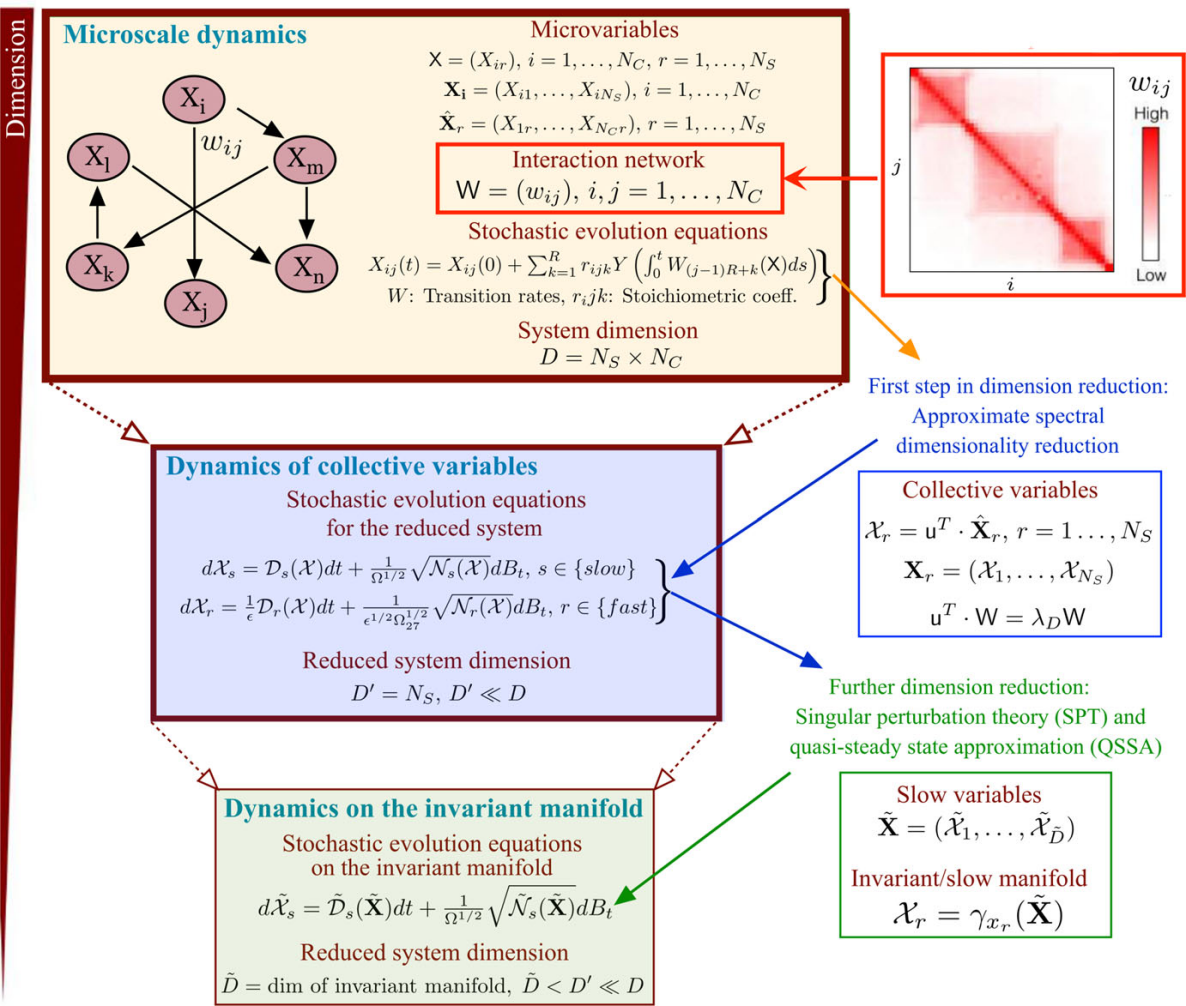
Schematic representation of the dimension reduction procedure. We undertake the dimension reduction procedure in two steps. We start by applying a stochastic generalization of the spectral reduction method put forward in Laurence et al. (2019). The connectivity matrix (in red), $\textsf{W}$, provides the strength of the contacts between the chromatin sub-regions that accounts for the 3D structure of the portion of chromatin. In the first step (in blue), we use spectral dimension reduction to reduce the dimensionality of the system from $N_C\times N_S$ to $N_S$. The second step (green) involves exploiting separation of time scales by using singular perturbation analysis (Bruna et al. 2014; Alarcón et al. 2021). This step reduces the dimension of the system from $N_S$ to the dimension of the (mean-field) invariant manifold, $\tilde{D}$. In general, $\tilde{D}\ll N_C\times N_S$. In the specific case of the system tackled in this work, $\tilde{D}=2$ (full details are provided in this SI Appendix).

The pipeline for dimension reduction of our stochastic model is shown in Fig. 2. For clarity, we split its description into two parts.

The first step is based upon a dimension reduction method that reduces high-dimensional dynamical systems, such as Eqs. (5)-(6), to a low-dimensional version, which can then be used to describe the collective dynamics of the original system. The dimension reduction method is based on spectral graph theory, particularly on the dominant eigenvalue and eigenvector of the matrix that defines the connectivity pattern of the system (Laurence et al. 2019; Vegué et al. 2023).

The second step involves the quasi-steady-state approximation (QSSA), Bruna et al. (2014); Alarcón et al. (2021), also known as the stochastic averaging principle, which provides a practical way to reduce the number of degrees of freedom in the system. The key parameter that determines the form of the reduced model is the ratio of the timescale for fast variables to the timescale for the evolution of the slow variables. In our case, we apply the QSSA to the equations for the collective dynamics (coarse-grained system) derived from the spectral dimension reduction.

#### Spectral dimension reduction of the stochastic microscopic system

Spectral dimension reduction (SDR) is a procedure that was introduced in Gao et al. (2016) and further developed in Laurence et al. (2019); Vegué et al. (2023) as a method to coarse-grain the dynamics of deterministic, high-dimensional dynamical systems running on complex networks. Let $\textsf{X}=(X_{ij})$ where $i=1,\dots ,N_S$ and $j=1,\dots ,N_C$. $N_C$ and $N_S$ are the number of nodes in the network and the number of dynamical variables, respectively, so that the dimension of the dynamical system, *D*, is $D=N_S\times N_C$. The (deterministic) SDR method (Laurence et al. 2019) consists of considering a vector of weights $\textsf{u}=\{u_i\}_{i=1,\dots ,N_C}$ and a set of collective (or averaged) variables ${\mathcal X}_i=\sum _ju_jX_{ij}$.

For a deterministic system with $N_S=1$, Laurence et al. (2019) considered the set of ODEs $\dot{x}_i=f(x_i)+\sum _{j=1}^{N_C}w_{ij}F(x_i,x_j)$. The SDR method posits that such a dynamical system on a homogeneous network can be reduced to the dynamics of the corresponding collective variable for an appropriate choice of the weights $u_i$. To do so, the method proceeds to make a Taylor expansion of the functions *f* and *F* around ${\mathcal X}$ and $(\Phi _1{\mathcal X},{\Phi _2}{\mathcal X})$, respectively. It can be shown that, to the lowest order, the dynamics of the collective variable ${\mathcal X}$ is given by $\dot{{\mathcal X}}=|\textsf{u}|_1f\left( \frac{{\mathcal X}}{|\textsf{u}|_1}\right) +\alpha _w F\left( \Phi _1^*{\mathcal X},\frac{{\mathcal X}}{|\textsf{u}|_1}\right)$. The SDR method allows us to determine in a self-consistent way the quantities $\textsf{u}$, $\alpha _w$, and $\Phi _1^*$, in terms of the spectral properties of the matrix $\textsf{W}$.

Here, we extend the SDR method to a Markov-jump process (MJP). As a result, we obtain a set of stochastic differential equations (SDEs) for the collective variables $\{\mathcal {X}_i\}_{i=1}^{N_S}$, thereby reducing the dimension of the problem from $D=N_S\times N_C$ to $D^{\prime }=N_S\ll D$.

Consider Eqs. (5)-(6) over the time interval $(t,t+dt]$ with $dt\ll 1$. Under the conditions established by the Chemical Langevin Equation (Gillespie 2000), we can write:

$$\begin{aligned} x_{ij}(t+dt)=x_{ij}(t)+\sum _{k=1}^Rr_{ijk}\frac{1}{\Omega _{27}}Y\left( \Omega _{27}\omega _{(j-1)R+k}(\textsf{x})dt\right) ,\;i=1,2,3 \end{aligned}$$

$$\begin{aligned} x_{ij}(t+dt)=x_{ij}(t)+\sum _{k=1}^Rr_{ijk}\frac{1}{\Omega _{E}}Y\left( \frac{1}{\epsilon }\Omega _{E}\omega _{(j-1)R+k}(\textsf{x})dt\right) ,\;i=4,\dots ,N_S. \end{aligned}$$

The stochastic dynamics of the collective variables are thus formally given by:

$$\begin{aligned} {\mathcal X}_{i}(\tau +d\tau )={\mathcal X}_{i}(\tau )+\sum _{j=1}^{N_C}\sum _{k=1}^Ru_jr_{ijk}\frac{1}{\Omega _{27}}Y\left( \Omega _{27}\omega _{(j-1)R+k}(\textsf{x})d\tau \right) ,\;i=1,2,3 \end{aligned}$$

$$\begin{aligned} {\mathcal X}_{i}(\tau +d\tau )={\mathcal X}_{i}(\tau )+\sum _{j=1}^{N_C}\sum _{k=1}^Ru_jr_{ijk}\frac{1}{\Omega _{E}}Y\left( \frac{1}{\epsilon }\Omega _{E}\omega _{(j-1)R+k}(\textsf{x})d\tau \right) ,\;i=4,\dots ,N_S. \end{aligned}$$

Since $Y(\lambda )\sim \text{ Poisson }(\lambda )$, the random variables $Z_{(j-1)R+k}(\Omega )\equiv u_jr_{ijk}Y(\Omega \omega _{(j-1)R+k}(\textsf{x})dt)$ are such that their average and variance are given by $m_{(j-1)R+k}=u_jr_{ijk}\Omega \omega _{(j-1)R+k}(\textsf{x})dt$ and $\sigma ^{2}_{(j-1)R+k}=u_i^2r_{ijk}^2\Omega \omega _{(j-1)R+k}(\textsf{x})$, respectively. Recalling general results regarding the central limit theorem for such variables (see Loève Loève 1977), we have that:

$$\begin{aligned} \frac{\sum _{j=1}^{N_C}Z_{(j-1)R+k}(\Omega )-\sum _{j=1}^{N_C}m_{(j-1)R+k}}{\sqrt{\sum _{j=1}^{N_C}\sigma _{(j-1)R+k}^2}}\rightarrow G(0,1) \end{aligned}$$

as $N_C\rightarrow \infty$ where *G*(0, 1) stands for a standard Gaussian random variable. Using this result and taking $dt\rightarrow 0$, the stochastic equations for $\mathcal {X}_i$ can be rewritten as:

7 $$\begin{aligned} d{\mathcal X}_{i}= &  \left( \sum _{j,k}u_jr_{ijk}\omega _{(j-1)R+k}(\textsf{x})\right) dt\nonumber \\ &  +\frac{1}{\Omega _{27}^{1/2}}\sqrt{\sum _{j,k}u_i^2r_{ijk}^2\omega _{(j-1)R+k}(\textsf{x})}dB_{\tau },\;i=1,2,3 \end{aligned}$$

8 $$\begin{aligned} d{\mathcal X}_{i}= &  \frac{1}{\epsilon }\left( \sum _{j,k}u_jr_{ijk}\omega _{(j-1)R+k}(\textsf{x})\right) dt\nonumber \\ &  +\frac{1}{\epsilon ^{1/2}\Omega _{E}^{1/2}}\sqrt{\sum _{j,k}u_i^2r_{ijk}^2\omega _{(j-1)R+k}(\textsf{x})}dB_{\tau },\;i=4,\dots ,N_S. \end{aligned}$$

where $dB_t=G(0,dt)$ i.e., it corresponds to an uncorrelated white noise with a Gaussian distribution such that $\langle dB_t\rangle =0$ and $\langle dB_t^2\rangle =dt$.

In order to proceed forward, we note that, provided the form of the transition rates (see Table 2), the drift and diffusion terms can be written as Laurence et al. (2019):

$$\begin{aligned} \sum _{j,k}u_jr_{ijk}\omega _{(j-1)R+k}(\textsf{x})=\sum _{j}u_j\left( f_i(\textsf{x}_j)+\sum _{k=1}^{R}\sum _{l=1}^{N_C}w_{jl}F_{i,k}(x_{ij},x_{kl})\right) \end{aligned}$$

$$\begin{aligned} \sum _{j,k}u^2_jr_{ijk}^2\omega _{(j-1)R+k}(\textsf{x})=\sum _{j}u^2_j\left( g_i(\textsf{x}_j)+\sum _{k=1}^{R}\sum _{l=1}^{N_C}w_{jl}G_{i,k}(x_{ij},x_{kl})\right) \end{aligned}$$

where $\textsf{x}_j=\{x_{ij}\}_{i=1}^{N_S}$ and $j\in [1,N_C]$. Furthermore, $F_{i,k}(y,z)\propto yz$ and $G_{i,k}(y,z)\propto yz$ (see Eqs. (1)-(4) and Table 3).

The spectral reduction method proceeds as follows. Consider first the functions $f_i$ and $F_{i,k}$ and their Taylor expansions around $\textsf{x}_j=\phi {\mathcal X}$, and $x_{ij}=\Phi _1{\mathcal X}_{i}$ and $x_{kl}=\Phi _2{\mathcal X}_k$, respectively. The SDR method (Laurence et al. 2019) posits that, under suitable conditions imposed on the contact matrix, $\textsf{W}$, enshrined in the Perron-Fobrenius theorem (Gantmacher 1963), one can find conditions for $\textsf{u}$, $\phi$, $\Phi _1$, and $\Phi _2$ under which the first order contributions vanish identically, so that

9 $$\begin{aligned} \mathcal {D}_i(\mathcal {X})=\sum _{j,k}u_jr_{ijk}\omega _{(j-1)R+k}(\textsf{x})=|u|_1f_i\left( \frac{{\mathcal X}}{|u|_1}\right) +\alpha _w\sum _kF_{i,k}\left( \Phi _1^*{\mathcal X}_i,\frac{{\mathcal X}_k}{|\textsf{u}|_1}\right) +{\mathcal O}(\Delta ^2) \end{aligned}$$

where, $\mathcal {D}_i(\mathcal {X})$ will be referred to as the coarse-grained drift, $\textsf{u}$ is the dominant eigenvector of $\textsf{W}^T$, $\phi =\Phi _2=|u|_1^{-1}$, with $|u|_1=\sum _{j=1}^{N_C}u_j$, $\alpha _w=\sum _{i,j=1}^{N_C}w_{ij}u_j$, $\Phi _1^*=\frac{\textsf{u}^T\cdot \textsf{K}\cdot \textsf{u}}{\alpha _w\textsf{u}^T\cdot \textsf{u}}$. These conditions leading to these quantities are derived in detailed in Appendix C.

By proceeding similarly with functions $f_i$ and $F_{i,k}$, we can derive an expression for the coarse-grained noise, $\mathcal {N}_i(\mathcal {X})$, and the conditions of $\gamma$, $\Gamma _1$, and $\Gamma _2$ so that the first order corrections vanish:

10 $$\begin{aligned} \mathcal {N}_i(\mathcal {X})=\sum _{j,k}u^2_jr^2_{ijk}\omega _{(j-1)R+k}(\textsf{x})=\Vert u\Vert ^2g_i\left( \frac{{\mathcal X}}{\Vert u\Vert ^2}\right) +\alpha _w\sum _kG_{i,k}\left( \Gamma _1^*{\mathcal X}_i,\frac{{\mathcal X}_k}{|\textsf{u}|_1}\right) +{\mathcal O}(\Delta ^2) \end{aligned}$$

where $\gamma =\left( \Vert u\Vert ^2\right) ^{-1}$ with $\Vert u\Vert ^2=\sum _{i,j=1}^{N_C}u_j^2$, $\Gamma _1^*=\frac{\textsf{u}^T\cdot \textsf{K}\cdot \textsf{v}}{\alpha _w\textsf{u}^T\cdot \textsf{u}}$, and $\Gamma _2=|u|_1^{-1}$.

With these results, we can finally write the set of SDEs governing the dynamics of the collective variables, ${\mathcal X}=\{{\mathcal X}_i\}_{i=1\dots ,N_S}$

11 $$\begin{aligned} &  d{\mathcal X}_i={\mathcal D}_i({\mathcal X})dt+\frac{1}{\Omega _{27}^{1/2}}\sqrt{{\mathcal N}_i({\mathcal X})}dB_t,\;i=1,2,3 \end{aligned}$$

12 $$\begin{aligned} &  d{\mathcal X}_i=\frac{1}{\epsilon }{\mathcal D}_i({\mathcal X})dt+\frac{1}{\epsilon ^{1/2}\Omega _{27}^{1/2}}\sqrt{{\mathcal N}_i({\mathcal X})}dB_t,\;i=4,\dots ,N_S \end{aligned}$$

where $\mathcal {D}_i(\mathcal {X})$ and $\mathcal {N}_i(\mathcal {X})$ are given by Eqs. (9) and (10), respectively.

This first step in our dimension redcution procedure reduces the dimension of the stochastic dynamical system from $D=N_S\times N_C$ to $D^{\prime }=N_S$.

Finally, this reduction is not unique, as any pair of eigenvalue/eigenvector such that $u_i>0$ could be used. However, the natural choice is that pair corresponding to the dominant eigenvector, as it is the one that bears the strongest influence of the structure on the dynamics, particularly for networks with a large spectral gap (i.e., networks with no modular structure). Although in general there is no reason to assume that the dominant eigenvalue and eigenvector be real, there is a large class of systems for which we know that they are real. For example, strongly connected networks with non-negative weights are within this category as per the Perron-Frobenius theorem (Gantmacher 1963).

#### Multi-scale singular perturbation analysis

To reduce the dimension of the system even further, we exploit the separation of time scales in the coarse-grained system for the collective variables, Eqs. (11)-(12) (see also Appendix A). In this Section, we use the singular perturbation method to find a FPE only for the slow variables. This method allows us to eliminate the dependency on the fast variables. Such elimination proceeds in two steps. First, we find the so called inner solution, where the slow variables are considered as frozen and we can compute the PDF for the fast variables. In the second step, we compute the outer solution (or equation), whereby we average out the fast variables, leading to an evolution equation for the slow variables.

In order to perform our multiple-scale analysis and carry out the last step in our dimension reduction method, we write the Fokker-Planck (FP, forward Kolmogorov) equation corresponding to the system of SDEs, Eqs. (11)-(12), which describes the time evolution of the probability density function (PDF), $\Psi ({\mathcal X},\tau )$:

$$\begin{aligned} \partial _{\tau }\Psi ({\mathcal X},\tau )=\sum _{slow}{\mathcal L}_i^{(s)}[\Psi ]+\frac{1}{\epsilon }\sum _{fast}{\mathcal L}_i^{(f)}[\Psi ], \end{aligned}$$

where the operators ${\mathcal L}_i^{(s)}$ and ${\mathcal L}_i^{(f)}$ are given by:

$$\begin{aligned} {\mathcal L}_i^{(s)}[\Psi ]=-\partial _{{\mathcal X}_i}\left( {\mathcal D}_i\Psi \right) +\frac{1}{2\Omega _{27}}\partial ^2_{{\mathcal X}_i}\left( {\mathcal N}_i\Psi \right) , \end{aligned}$$

$$\begin{aligned} {\mathcal L}_i^{(f)}[\Psi ]=-\partial _{{\mathcal X}_i}\left( {\mathcal D}_i\Psi \right) +\frac{1}{2\Omega _{E}}\partial ^2_{{\mathcal X}_i}\left( {\mathcal N}_i\Psi \right) , \end{aligned}$$

respectively, where $\mathcal {D}_i$ and $\mathcal {N}_i$ are given by Eqs. (9) and (10).

In order to proceed forward, we propose that the PDF $\Psi ({\mathcal X},\tau )$ can be expanded in powers of $\epsilon$: $\Psi =\Psi _0+\epsilon \Psi _1+{\mathcal O}(\epsilon ^2)$ (Horsthemke and Lefever 2006; Bruna et al. 2014). By using such an expansion, it is straightforward to find that $\Psi _0$ and $\Psi _1$ satisfy:

13 $$\begin{aligned} &  \sum _{fast}{\mathcal L}_i^{(f)}[\Psi _0]=0, \end{aligned}$$

14 $$\begin{aligned} &  \sum _{fast}{\mathcal L}_i^{(f)}[\Psi _1]=\partial _{\tau }\Psi _0-\sum _{slow}{\mathcal L}_i^{(s)}[\Psi _0]. \end{aligned}$$

where sum over *fast* refers to sum over the set of fast variables, $i\in [4,N_S]$. We now proceed to solve Eqs. (13) and (14) order by order. Let $\mathcal {X}_s\equiv \{\mathcal {X}_i\}_{i=1}^3$ and $\mathcal {X}_f\equiv \{\mathcal {X}_i\}_{i=4}^{N_S}$. Regarding $\Psi _0({\mathcal X},\tau )$, by inspection of Eq. (13) and the corresponding drift and noise terms (see Appendix D), $\Psi _0$ can be assumed to have the form $\Psi _0({\mathcal X},\tau )=\psi _s({\mathcal X}_s,\tau )\prod _{i=4}^{N_S}\psi _{i}({\mathcal X}_i\vert {\mathcal X}_s)$. In what follows, to simplify the notation, we will omit the *conditioned to* ${\mathcal X}_s$ when we refer to $\psi _i$. Then Eq. (13) becomes:

$$\begin{aligned} \psi _s({\mathcal X}_s,\tau )\left( \sum _{i\in fast}\left( \prod _{j\ne i}\psi _{j}({\mathcal X}_j)\right) {\mathcal L}_i^{(f)}[\psi _i({\mathcal X}_i)]\right) =0, \end{aligned}$$

which has nontrivial solutions if ${\mathcal L}_i^{(f)}[\Psi _i({\mathcal X}_i)]=0\phantom {m} \forall i\in [4,N_S]$. Using the conservation laws Eqs. (C12)-(C15), they reduce to four one-dimensional, steady-state FP equations, for which a particular solution can be found by integrating by quadratures. Since ${\mathcal L}_i^{(f)}[\Psi _i({\mathcal X}_i)]=-\partial _{{\mathcal X}_i}{\mathcal J}_i^{(f)}[\Psi _i]$, we can set ${\mathcal J}_i^{(f)}[\Psi _i]=0$:

15 $$\begin{aligned} \psi _i({\mathcal X}_i)=\frac{\textbf{N}_i}{{\mathcal N}_i}\exp \left( 2\Omega _E\int ^{{\mathcal X}_i}\frac{{\mathcal D}_i}{{\mathcal N}_i}ds\right) , \end{aligned}$$

where $\textbf{N}_i$ are the normalization constants so that $\int \psi _id{\mathcal X}_i=1$. The drift, ${\mathcal D}_i$, and noise, ${\mathcal N}_i$, terms are given in Appendix D.

Equation (13) provides no information on $\psi _s({\mathcal X}_s,\tau )$ and thus we need to resort to the first order, Eq. (14), in order to determine it. When tackling Eq. (14), we find a well-known problem in singularly perturbed problems. Since $\sum _{fast}{\mathcal L}_i^{(f)}[\Psi _0]=0$ has non-trivial solutions, for the corresponding inhomogeneous problem Eq. (14) to have solution(s), its right-hand side needs to satisfy additional conditions (Stakgold 1998). Such conditions are known as solvability conditions, and they eventually provide the equation for determining $\Psi _0$ (Stakgold 1998; Ward 1999; Bruna et al. 2014; Kogan et al. 2014). Consider $\varphi ({\mathcal X}_f)$ defined as:

$$\begin{aligned} \varphi ({\mathcal X}_f)=\left\{ \begin{array}{l}1 \text{ if } {\mathcal X}_f\in \textbf{D},\\ 0 \text{ otherwise, }\end{array}\right. \end{aligned}$$

where $\textbf{D}=[0,|u|_1e_0/\sqrt{N_c}]\times [0,|u|_1u_0/\sqrt{N_c}]\times [0,|u|_1h_0/\sqrt{N_c}]\times [0,|u|_1s_0/\sqrt{N_c}]$, and consider also its scalar product with the left hand side of Eq. (14):

$$\begin{aligned} \langle \varphi ,\sum _{fast}{\mathcal L}_i^{(f)}[\Psi _1]\rangle =\langle \sum _{fast}{\mathcal L}_i^{(f)^{\dagger }}[\varphi ],\Psi _1\rangle =0, \end{aligned}$$

which follows in a straightforward way since ${\mathcal L}_i^{(f)^{\dagger }}[\psi ]={\mathcal D}_i\partial _{{\mathcal X}_i}\psi +\frac{1}{2\Omega _E}{\mathcal N}_i\partial ^2_{{\mathcal X}_i}\psi$ (Oksendal 2003). The scalar product is defined by:$\langle \varphi ,\phi \rangle =\int _{\textbf{D}}\varphi \phi d{\mathcal X}_f$. The solvability condition thus reads:

$$\begin{aligned} 0=\int _{\textbf{D}}\partial _{\tau }\Psi _0d{\mathcal X}_f-\int _{\textbf{D}}\sum _{slow}{\mathcal L}_{i}^{(s)}[\Psi _0]d{\mathcal X}_f. \end{aligned}$$

Taking into account that $\Psi _0({\mathcal X},\tau )=\psi _s({\mathcal X}_s,\tau )\prod _{fast}\psi _{i}({\mathcal X}_i)$, this condition leads to:

16 $$\begin{aligned} \partial _{\tau }\psi _s=\langle {\mathcal L}_s\rangle [\psi _s], \end{aligned}$$

where

$$\begin{aligned} \langle {\mathcal L}_s\rangle [\psi _s]=\int _{\textbf{D}}\sum _{slow}{\mathcal L}_j^{(s)}[\psi _s]\left( \prod _{fast}\psi _i({\mathcal X}_i)\right) d{\mathcal X}_s=\sum _{slow}-\partial _{{\mathcal X}_i}\langle {\mathcal D}_i\rangle \psi _s+\frac{1}{2\Omega _{27}}\partial ^2_{{\mathcal X}_i}\langle {\mathcal N}_i\rangle \psi _s. \end{aligned}$$

with $$\langle {\mathcal D}_i\rangle =\int _{\textbf{D}}{\mathcal D}_i\left( \prod _{fast}\psi _j({\mathcal X}_j)\right) d{\mathcal X}_s$$ and $$\langle {\mathcal N}_i\rangle =\int _{\textbf{D}}{\mathcal N}_i\left( \prod _{fast}\psi _j({\mathcal X}_j)\right) d{\mathcal X}_s$$.

In order to provide an analytic expression of the averaged operator $\langle {\mathcal L}_s\rangle$, we recall the form of the PDF, Eqs. (15). Their exponential form and the fact that we are considering the situation where $\Omega _E>>1$ (with $\Omega _{27}>>\Omega _E$) allows us to obtain an asymptotic approximation of the integrals involved in $\langle {\mathcal D}_i\rangle$ and $\langle {\mathcal N}_i\rangle$ using Laplace’s method (Murray 1984). Under such an approximation, to the lowest order, we have

$$\begin{aligned} \langle {\mathcal D}_i\rangle ={\mathcal D}_i({\mathcal X}_s,{\mathcal X}^*_f),\;\langle {\mathcal N}_i\rangle ={\mathcal N}_i({\mathcal X}_s,{\mathcal X}^*_f),\;i\in slow \end{aligned}$$

where

$$\begin{aligned} {\mathcal X}_j^*=\min _{{\mathcal X}_j} {\mathcal U}_j({\mathcal X}_j),\;j\in fast \end{aligned}$$

with ${\mathcal U}_j({\mathcal X}_j)=-\int _0^{{\mathcal X}_j}\frac{{\mathcal D}_j}{{\mathcal N}_j}ds$, $j\in fast$. ${\mathcal X}^*_j$ are the roots of ${\mathcal D}_j=0$, and therefore correspond to the steady states of the mean-field system. The analytical expression for the averaged drift and noise terms associated with the slow collective variables ${\mathcal X}_1$ (methylation) and ${\mathcal X}_2$ (acetylation) are given in Section Appendix D

### Robustness and Parameter Sensitivity Analysis: A WKB Approach

The analysis of tipping points and their associated early warning signals requires means to study the sensitivity of the system to changes in parameter values, specifically regarding the existence of bifurcations or phase transitions (Xu et al. 2021). It is not uncommon for such efforts to be seriously hindered in complex, high-dimensional systems. In Section 2.2, we have developed a dimension reduction methodology that allows us to coarse-grain the stochastic system responsible for EL maintenance into a low-dimensional system. To complete our analysis, we must obtain an analytical (approximation of the) solution of low-dimensional FPE, Eq. (16). To proceed further, we will assume that the system has reached a steady state, so that $\psi _s$ satisfies:

17 $$\begin{aligned} \langle {\mathcal L}_s\rangle [\psi _s]=0. \end{aligned}$$

Furthermore, since we are also assuming that $\Omega _{27}\gg 1$, we use WKB asymptotics to solve for $\psi _s$. The WKB method has been used extensively to analyse FPEs (see Cohen and Lewis 1967; Maslov and Fedoriuk 1981; Hinch and Chapman 2005; Bressloff 2014; Bonnemain and Ullmo 2019 for some exmples). Here we provide an account of the methodology, complemented by detailed derivations in the Supplementary Materials. WKB asymptotics proceeds by making the following Ansatz regarding $\psi _s$:

$$\begin{aligned} \psi _s\sim \mathcal {R}(\mathcal {X}_s)e^{\Omega _{27}\mathcal {S}(\mathcal {X}_s)}. \end{aligned}$$

WKB asymptotics prescribe that both $\mathcal {R}$ and $\mathcal {S}$ be expanded in powers of $\Omega _{27}^{-1}$: $\mathcal {S}=\mathcal {S}_0+\sum _{n\ge 1}\Omega _{27}^{-n}\mathcal {S}_n$ and $\mathcal {R}=\mathcal {R}_0+\sum _{n\ge 1}\Omega _{27}^{-n}\mathcal {R}_n$. To the lowest order $\psi _s\sim e^{\Omega _{27}\mathcal {S}_0+\log \mathcal {R}_0+\mathcal {O}(\Omega _{27}^{-1})}$, which implies that the shape of $\psi _s$ is strongly dominated by $\mathcal {S}_0$.

According to the WKB methodology, $\mathcal {S}_0$ satisfies a Hamilton-Jacobi equation (Bonnemain and Ullmo 2019) (see also the Supplementary Materials):

$$\begin{aligned} \langle \mathcal {H}\rangle \left( \mathcal {S}_0,\partial _{\mathcal {X}_s}\mathcal {S}_0\right) =0, \end{aligned}$$

where

$$\begin{aligned} \langle {\mathcal H}\rangle ({\mathcal X}_s,{\mathcal P}_s)=\sum _{i\in slow}\left( -\langle {\mathcal D}_i\rangle {\mathcal P}_i+\frac{1}{2}\langle {\mathcal N}_i\rangle {\mathcal P}_i^2\right) . \end{aligned}$$

so that

18 $$\begin{aligned} \mathcal {S}_0(\mathcal {X}_s)=\int _{\mathcal {X}_0}^{\mathcal {X}_s}\mathcal {P}\cdot d\mathcal {X}, \end{aligned}$$

where $\mathcal {X}$ and $\mathcal {P}$ are the solutions of the Hamilton equations

19 $$\begin{aligned} \frac{d{\mathcal X}_i}{ds}=-\partial _{{\mathcal P}_i}\langle {\mathcal H}\rangle ;\; \frac{d{\mathcal P}_i}{ds}=\partial _{{\mathcal X}_i}\langle {\mathcal H}\rangle , \end{aligned}$$

with initial conditions ${\mathcal X}_s(s=0)={\mathcal X}_0$ and ${\mathcal P}_s(s=0)={\mathcal P}_0$. Because of this mathematical structure, $\mathcal {S}_0$ is referred to as the *action* and plays a role similar to that of a quasi-potential (Xu et al. 2021).

#### Robustness in bistable systems

We will now focus our analysis on regimes where the system exhibits bistability and study the robustness of the stable steady-states (SSSs). Within the context of the WKB approximation, we will analyze the resilience of SSSs to changes in parameter values. We introduce an approach to perform sensitivity analysis without the need for extensive parameter sweeps, which are computationally costly and often impractical. We will denote by $\Theta \in R^{D_p}$ the vector whose components are the kinetic parameters of our model. $D_p$ is the number of such parameters. Specifically, we will tackle the robustness of hypoacetylated states. Let $\mathcal {X}_0$ be the coordinates of the unstable saddle point and $\mathcal {X}_{\pm }$, the coordinates of the stable nodes. In this context, we use as a proxy for the robustness of $\mathcal {X}_{\pm }$ the quantity $\Delta {\mathcal S}_{\pm }(\Theta )=\mathcal {S}_0(\mathcal {X}_{\pm })-\mathcal {S}_0(\mathcal {X}_{0})=\int _{\mathcal {X}_{0}}^{\mathcal {X}_{\pm }}\mathcal {P}\cdot d\mathcal {X}$. This quantity captures the features of the quasi-potential landscape associated with the FPE, Eq. (17). Specifically, it quantifies the height of the quasi-potential barrier the system needs to overcome to undergo a (noise-induced) transition from $\mathcal {X}_{\pm }$ to $\mathcal {X}_{\mp }$. $\Delta {\mathcal S}_{\pm }$ thus provides a direct measure of the robustness of the corresponding equilibrium to intrinsic noise, and therefore, a measure of canalization of the associated epigenetic landscapes. It is therefore central to our analysis to ascertain what system parameters are those to which $\Delta {\mathcal S}_{\pm }(\Theta )$ exhibits a higher degree of sensitivity. The natural measure of sensitivity is their gradient in parameter space:

20 $$\begin{aligned} \nabla _{\Theta }\Delta \mathcal {S}_{\pm }(\Theta _*)=\left( \partial _{\theta _1}\Delta \mathcal {S}_{\pm },\dots ,\partial _{\theta _N}\Delta \mathcal {S}_{\pm }\right) ^T|_{\Theta =\Theta _*}. \end{aligned}$$

The sign and magnitude of each component of this vector provide the necessary information regarding which parameters increase the robustness of the EL and which ones decrease it. The details of the computation of this quantity are given in the Supplementary Materials.

## Results

### Coarse-Grained Equations

The microscopic model has been coarse-grained into the following two-dimensional system of SDEs (see Section 2.2 and Appendix C and D for details):

21 $$\begin{aligned} &  d\mathcal {X}_1=\langle \mathcal {D}_1\rangle dt+\frac{1}{\Omega _{27}^{1/2}}\sqrt{\langle \mathcal {N}_1\rangle }dB_t, \end{aligned}$$

22 $$\begin{aligned} &  d\mathcal {X}_2=\langle \mathcal {D}_2\rangle dt+\frac{1}{\Omega _{27}^{1/2}}\sqrt{\langle \mathcal {N}_2\rangle }dB_t,\end{aligned}$$

23 $$\begin{aligned} &  \mathcal {X}_3=|\textsf{u}|_1s_0-\mathcal {X}_1-\mathcal {X}_2, \end{aligned}$$

where

$$\begin{aligned} &  \langle \mathcal {D}_1\rangle =\frac{k_3k_1\frac{e_0}{\sqrt{N_C}}|\textsf{u}|_1(\alpha _1+\frac{\alpha _w\Phi _1^*}{|\textsf{u}|_1}{\mathcal X}_1){\mathcal X}_3}{k_2+k_3+k_1|\textsf{u}|_1(\alpha _1+\frac{\alpha _w\Phi _1^*}{|\textsf{u}|_1}{\mathcal X}_1){\mathcal X}_3}\\ &  -\frac{k_6k_4\frac{u_0}{\sqrt{N_C}}|\textsf{u}|_1(\alpha _4+\frac{\alpha _w\Phi _1^*}{|\textsf{u}|_1}{\mathcal X}_2){\mathcal X}_1}{k_5+k_6+k_4|\textsf{u}|_1(\alpha _4+\frac{\alpha _w\Phi _1^*}{|\textsf{u}|_1}{\mathcal X}_2){\mathcal X}_1}, \\ &  \langle \mathcal {D}_2\rangle =\frac{k_9k_7\frac{h_0}{\sqrt{N_C}}|\textsf{u}|_1(\alpha _7+\frac{\alpha _w\Phi _1^*}{|\textsf{u}|_1}{\mathcal X}_2){\mathcal X}_3}{k_8+k_9+k_7|\textsf{u}|_1(\alpha _7+\frac{\alpha _w\Phi _1^*}{|\textsf{u}|_1}{\mathcal X}_2){\mathcal X}_3}\\ &  -\frac{k_{12}k_{10}\frac{s_0}{\sqrt{N_C}}|\textsf{u}|_1(\alpha _{10}+\frac{\alpha _w\Phi _1^*}{|\textsf{u}|_1}{\mathcal X}_1){\mathcal X}_2}{k_{11}+k_{12}+|\textsf{u}|_1\frac{B}{\sqrt{N_C}}+k_{10}|\textsf{u}|_1(\alpha _{10}+\frac{\alpha _w\Phi _1^*}{|\textsf{u}|_1}{\mathcal X}_1){\mathcal X}_2}, \\ \langle \mathcal {N}_1\rangle = &  k_1\left( \left( \frac{2k_2+k_3}{k_2+k_3}\right) \alpha _1+\frac{\alpha _w}{|\textsf{u}|_1}\left( \Gamma _1^*+\frac{k_2}{k_2+k_3}\Phi _1^*\right) {\mathcal X}_1\right) e_2{\mathcal X}_3+\\ &  k_4\left( \left( \frac{2k_5+k_6}{k_5+k_6}\right) \alpha _4+\frac{\alpha _w}{|\textsf{u}|_1}\left( \Gamma _1^*+\frac{k_5}{k_5+k_6}\Phi _1^*\right) {\mathcal X}_2\right) e_U{\mathcal X}_1,\\ \langle \mathcal {N}_2\rangle = &  k_7\left( \left( \frac{2k_8+k_9}{k_8+k_9}\right) \alpha _7+\frac{\alpha _w}{|\textsf{u}|_1}\left( \Gamma _1^*+\frac{k_8}{k_8+k_9}\Phi _1^*\right) {\mathcal X}_2\right) e_H{\mathcal X}_3+\\ &  k_{10}\left( \left( \frac{2k_{11}+k_{12}}{k_{11}+k_{12}}\right) \alpha _{10}+\frac{\alpha _w}{|\textsf{u}|_1}\left( \Gamma _1^*+\frac{k_{11}}{k_{11}+k_{12}}\Phi _1^*\right) {\mathcal X}_1\right) e_S{\mathcal X}_2. \end{aligned}$$

The variables $\mathcal {X}_1$, $\mathcal {X}_2$, and $\mathcal {X}_3$ are the observables (weighted variables) corresponding to acetylated, methylated, and unmodified chromatin, respectively. A full description of the derivation of Eqs. (21)-(23) is given in the Supplementary Materials. Note that the form of the mathematical expressions of $\langle \mathcal {D}_1\rangle$ and $\langle \mathcal {D}_2\rangle$ match those of Eqs. (B2)-(B3), i.e., they are the addition of two Michaelis-Menten-like functions, one for addition and one for removal.

**Fig. 3:**
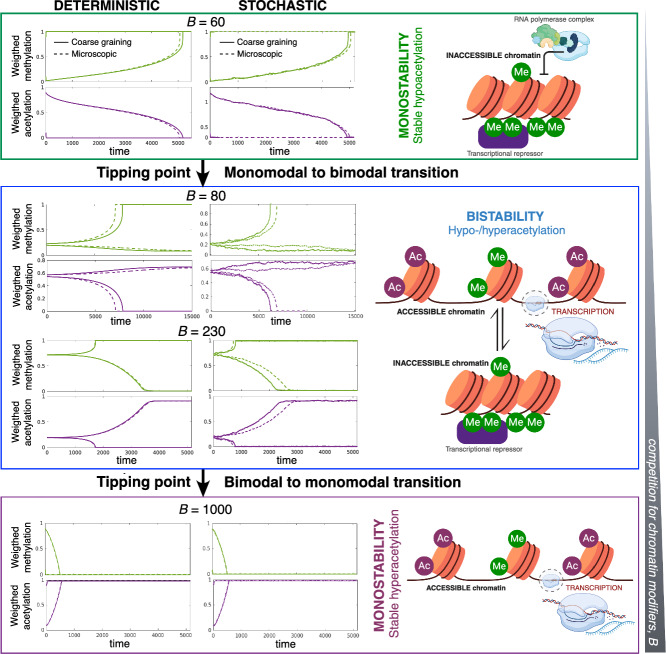
**Predictions of the coarse-grained (CG) dynamics match those of the microscopic (Micro.) system, specifically regarding tipping points.** Deterministic (first column) and stochastic (second column) simulations increasing the competition for chromatin modifiers, *B*. Predictions of the CG system (solid lines) predict with a high degree of accuracy the critical behavior (tipping points) of its microscopic counterpart (dashed lines). Panels display individual realizations using the all-to-all connectivity matrix. Parameter values are given in Supplementary Materials, Section IV.

Note that the right-hand side of Eq. (22) depends on the parameter *B* (see the expression for $\langle \mathcal {D}_2\rangle$ under Eq. (23)). As described in detail in Section 2.2, *B* is a proxy for the amount of sirtuins sequestered by substrates different from chromatin, e.g., DNA damage (Yang et al. 2023). It, therefore, quantifies the competition between chromatin and the other substrates of histone deacetylases (HDACs), specifically, sirtuins. Figure 3 shows numerical simulations comparing the predictions of the CG system with those of the microscopic dynamics with the all-to-all connectivity matrix. In these simulations, we have used increasingly larger values of *B*, corresponding to elevating the levels of competition for the HDACs. The predictions of the CG dynamics match those of the microscopic system, both in the mean-field, deterministic scenario and in the stochastic regime. A thorough comparison between the CG and the microscopic benchmark is provided in the Supplementary Materials, Figs. S3-S6.

**Fig. 4:**
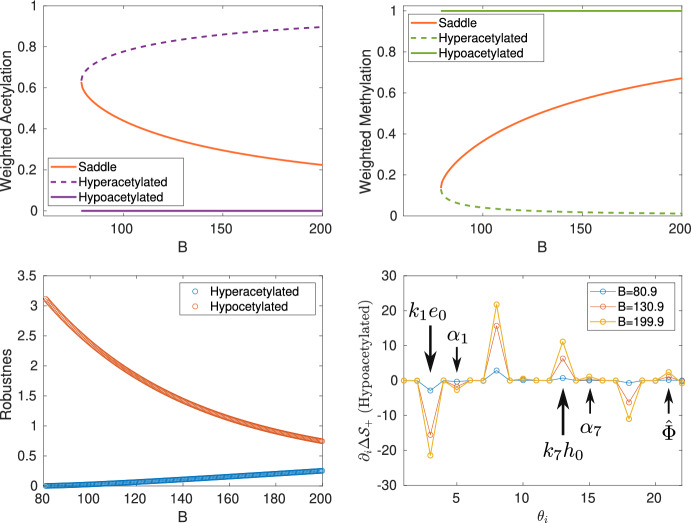
**Mean-field limit and stochastic coarse-grained (CG) approximations: tipping points and sensitivity analysis.** The deterministic limit of the CG approximation predicts that competition for histone deacetylases (quantified by the parameter *B*) induces tipping points in the stable ELs. The stochastic analysis allows us to quantify the robustness of the stable ELs and, more interestingly, the sensitivity of the robustness when the model parameters are changed. This sensitivity analysis allows us to determine which parameters are more effective at increasing or decreasing the robustness of the associated ELs. Legend: $\hat{\Phi }=\frac{\alpha _w\Phi ^*_1}{\vert \textsf{u}\vert _1}$. The quantities $\Delta \mathcal {S}_{\mp }$ defined in the main text are used as proxies for the robustness of hyper- and hypo-methylated states, respectively. The microscopic deterministic equations are given in Appendix B. This analysis has been done in the case of all-to-all connectivity. Parameter values are given in Supplementary Materials, Section IV.

### Competition for Chromatin Modifiers Triggers Tipping Points

The coarse-grained (CG) version of the epigenetic landscape model, Eqs. (21)-(23), allows us to analyze the system regarding the existence of transitions that alter the global structure of the landscape. Specifically, by analyzing the deterministic limit of the CG system, we address how competition for chromatin modifiers affects (HDACs in particular) the existence and stability of hypo- and hyper-acetylated landscapes. Such landscapes correspond to the steady-state observables $\mathcal {X}_-$ and $\mathcal {X}_+$, respectively. Our model predicts that as the level of competition for HDACs, *B*, increases, the landscape goes through several transitions (bifurcations). For intermediate values of *B*, the system is bistable, so that the hypo- and hyper-acetylated states coexist. As *B* decreases, the system approaches a tipping point (saddle-node bifurcation) where the hyperacetylated landscape ceases to exist (see Fig. 4). Conversely, if *B* increases, a second tipping point exists where the hypoacetylated state vanishes through a saddle-node bifurcation (see Fig. 3). It is noteworthy that the existence of the tipping points and the critical level of competition for HDAC (*B*) at which they occur predicted by the CG system are in excellent agreement with the results of the microscopic dynamics. These resuls are consitent with the experimental findings of Yang et al. (2023) regarding the effects of sirtuin sequestration by DNA double-strand breaks on the stability of and information contained in ELs, and also with recent computational results (Stepanova et al. 2025a).

### Robustness and Sensitivity Analysis

As discussed in the Section 2.3, the quantities $\Delta \mathcal {S}_{\mp }$ are proxies of the robustness of hyper- and hypo-methylated states, respectively, as long as we restrict our study of the epigenetic landscape (EL) to its bistable regime. Taking advantage of the low dimensionality of the stochastic coarse-grained representation, we use WKB asymptotics to compute the so-called action between the unstable (saddle) equilibrium and the stable (nodes) equilibria, corresponding to the hypo- and hyper-acetylated landscapes. By capturing the features of the quasi-potential landscape associated with the stochastic dynamics of the CG system, these quantities quantify the height of the quasi-potential barrier that the system has to overcome to leave the basin of attraction of one stable EL and move into the other.

Figure 4 shows that, as the system approaches the tipping point where the hyperacetylated state ceases to exist, its robustness tends to zero. By contrast, in the vicinity of such a transition, the robustness of the hypoacetylated landscape approaches its maximum value.

This notion of robustness allows us to explore the sensitivity of ELs to changes in specific parameters. In a nutshell (more details are given in the Materials & Methods), we can use Eq. (20), to identify the directions of the space of parameters in which the gradients of $\Delta \mathcal {S}_{\mp }$ are steeper. Such directions correspond to the parameters whose change produces larger variations in robustness. The result of this analysis is shown in Fig. 4. Not surprisingly, the steeper directions correspond to $k_1e_0$, $k_4u_0$, $k_7h_0$, and $k_{10}s_0$, i.e., to those parameters that are proportional to the total abundance of each of the chromatin modifiers (HMs, HDMs, HACs, and HDACs respectively).

Beyond this general observation, by looking specifically at the sensitivity to parameters related to the activity of HMs and HACs, one uncovers an interesting pattern. Besides the elevated sensitivity to $k_1e_0$ and $k_7h_0$, Fig. 4 shows that $\alpha _1$ and $\alpha _7$ (which correspond to the basal, unregulated, HM and HAC activity, respectively) are second in their effect on the robustness of hypoacetylated landscape. It is noteworthy that the robustness is also sensitive to the structure of the connectivity matrix $\textsf{W}$ (connectivity pattern, intensity of interactions, etc.), as revealed by the effect of $\hat{\Phi }$ on the robustness.

### The 3D Folding Structure of Chromatin Affects the Robustness of Hyper- and Hypo-acetylated Epigenetic Landscapes

**Fig. 5:**
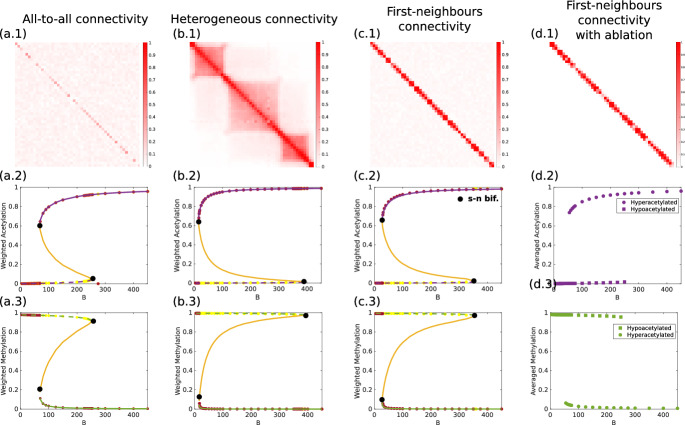
The CG method copes with different connectivity structures. We show results illustrating the robustness of our method to changes in connectivity structures. To carry out this analysis, we have used three different connectivities, namely, an all-to-all connectivity structure (a); a heterogeneous connectivity (b), as the ones detected in Hi-C measurements (see Fig. 2 in Szabo et al. (2019)); and a first-neighbors topology (c), including a case of first-neighbors with ablation (d). For simplicity, we have focused on the analysis of the mean-field limit system. Specifically, we have performed a bifurcation analysis with the parameter *B* as the control parameter. In all three cases, we show that the steady-state of the collective variables (weighted methylation and weighted acetylation) exhibits an excellent agreement between the microscopic dynamics (red and yellow dots) and the predictions of the coarse-grained system. Specifically, for different values of the DNA damage, *B*, we have let the microscopic system reach its steady state, i.e., $(m^*_i,a^*_i)_{i=1}^{N_S}$. We have then plotted the steady-state value of the average methylation and average acetylation, given by $\bar{M}=\frac{1}{N_S}\sum _{i=1}^{N_S} m^*_i\;\bar{A}=\frac{1}{N_S}\sum _{i=1}^{N_S} a^*_i$. Legend: dashed lines correspond to hypoacetylated landscapes and solid lines, to hyperacetylated landscapes. The black dots represent the occurrence of a saddle-node (s-n) bifurcation. The microsdopic deterministic equations are given in Appendix B. Details of the different patterns of connectivity are given in the Supplementary Materials, Section I. Parameter values are given in Supplementary Materials, Section IV.

Our sensitivity analysis (see Section 3.3) reveals an interesting property of the EL-generating system, namely, that the 3D folding structure of chromatin influences robustness. Specifically, the CG sensitivity analysis shows that variations of the parameter $\hat{\Phi }$, which is determined by both the structural features of the connectivity pattern and the strength of the interactions between sites, have a strong effect on the robustness of hypo- and hyper-acetylated ELs (see Fig. 4). To further explore this issue, we consider different connectivity patterns (see the Supplementary Materials, Section I for details of how the different patterns have been generated). For concreteness, we performed this analysis in the context of tipping induced by competition for sirtuins. Results of the bifurcation analysis for the mean-field limits (both microscopic and CG) are shown in Fig. 5(a-c). It is noteworthy that both the steady-state values and the critical behavior (tipping points) of the microscopic system are accurately captured by the CG system, regardless of the connectivity structure.

Furthermore, as we observe comparing Figs. 5(a), (b), and (c), the connectivity pattern associated with longer-range interactions corresponds to the narrowest bistability region. By contrast, the other two patterns, heterogeneous and first-neighbors exhibit wider bistability regions. Our results imply that the width of this region does not directly correlate with the robustness of bistability. However, our robustness analysis (see Fig. 4) proves that the parameter $\hat{\Phi }$, associated with the matrix $\textsf{W}$, influences robustness. Table 1 shows that $\hat{\Phi }$ is a predictor of the range in which bistability exists.

**Table 1:** $\hat{\Phi }$ **values.** We show the value of $\hat{\Phi }$ for the specific matrices used in Figs. 5(a), (b), and (c).

Connectivity patterns	$\hat{\Phi }$
All-to-all	4.7845
Heterogeneous	72.2203
First-neighbors	38.4737

Finally, our model also predicts that when the intensity of the connections between bins is either too strong or too weak, the ability of our system to exhibit bistable behavior is compromised (see Supplementary Materials Fig. S3).

### Exploring Epigenetic Tipping Points Under Realistic Heterogeneous Connectivity Patterns

We have considered a pattern of connectivity that reproduces the features observed in Hi-C interaction maps. At the scale of topologically associating domains, the interaction map is heterogeneous (Szabo et al. 2019; Lawson et al. 2023). It exhibits sub-regions that are tightly connected, while the connections between such sub-regions are much weaker or infrequent. It can thus be considered as an intermediate case between the first-neighbors and the all-to-all cases: the overall level of connectivity is much higher than in the first-neighbors pattern but not as dense as in the all-to-all connectivity. To analyze this case in the context of our CG methodology, we have generated a matrix (see Fig. 5(b)) with the features of that obtained from Hi-C data (see Fig. 2 in Szabo et al. (2019)). Using this heterogeneous connectivity pattern, we have derived the corresponding CG system and performed the bifurcation analysis to show that this connectivity pattern also supports tipping points induced by competition for HDAC. The results are shown in Fig. 5(b). As in the case of the other connectivity patterns (Figs. 5(a) and (c)), the CG approximation accurately predicts the critical behavior of the microscopic system. We conclude, therefore, that the CG method can be accurately applied to realistic heterogeneous connectivity patterns.

**Fig. 6:**
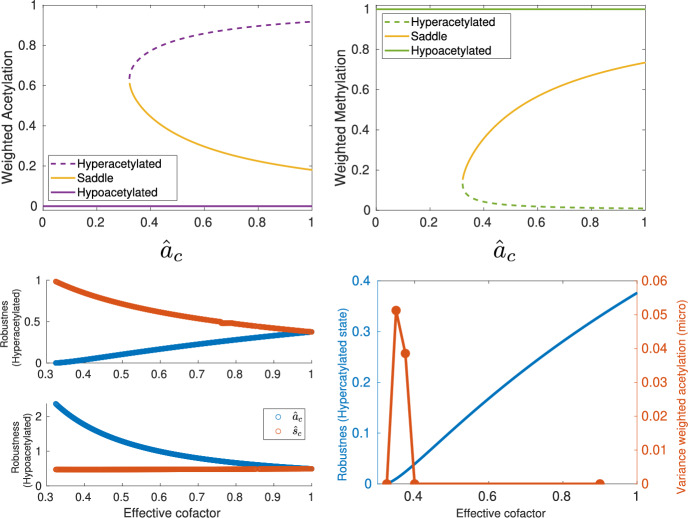
**Cofactors of chromatin modifiers as early warning indicators of tipping points in epigenetic landscapes.** The sensitivity analysis of the robustness of the stable ELs (see Fig. 4) indicates that the changes in the levels of metabolic cofactors SAM and AcoA (associated with the HMT and HDAC modifiers, respectively) alter the robustness of the stable ELs. Here we show that the mean-field limit of the CG approximation predicts a tipping point as AcoA availability decreases. The analysis of the stochastic CG approximation shows how the variation of these cofactors affects the robustness of the hyper- and hypo-acetylated ELs. Finally, we show that the predictions of the CG approximation accurately reproduce the behavior of the microscopic benchmark. This analysis has been done with the all-to-all connectivity. Parameter values are given in Supplementary Materials, Section IV.

### Bistable Chromatin Without Long-range Interactions

We have further pursued an *ablation study*, where we have used a pure, ablated first-neighbor (AFN) interaction pattern, i.e., one where $w_{ij}$ strictly equal to zero for all non-first-neighbor interactions (see Supplementary Materials, Section II). Since $\textsf{W}$ now has entries $w_{ij}=0$, the CG method cannot be applied, as now it is not granted that $\textsf{W}$ has a dominant eigenvector with all its components strictly positive (see Supplementary Materials). However, we can still solve numerically the microscopic dynamics, Eqs. (B2)-(B3). The results are shown in Fig. 5(d). Our simulations show that the system with the AFN matrix still exhibits bistability, although the bistability region becomes narrower when compared to the non-AFN first-neighbor chromatin architecture (cf. Fig. 5 (c.2) and (d.2)), suggesting that the latter’s background of low-intensity, long-range interactions stabilizes bistable chromatin. This is an interesting result regarding bistable chromatin. Most of the current models in the literature require long-range interactions as a necessary condition for bistability. The analysis with AFN interactions shows that our model does not require all-to-all or long-range interactions to support bistable chromatin. Since our model bins the genomic region of five nucleosomes in width (approximately), first-neighbor interactions in our model mean connections in the range of $\sim$750 bases. Our model requires no-longer range interactions for bistability.

### Metabolic Warning Signals

The sensitivity analysis provides a heterogeneous landscape of sensitivity, where some parameters (or combinations thereafter) are shown to affect deeply the robustness of the hyper- and hypo-acetylated states, whereas others barely have an effect (see Fig. 4). This analysis allows us to identify which parameters can synergise positively (i.e., facilitating the tipping point) or negatively (i.e., hindering the tipping point) with the competition for HDAC modifiers. Hereafter, we illustrate how our sensitivity analysis identifies candidates to be metabolic warning signals i.e., observable metabolic properties of the system that provide warning of an impending abrupt transition in the system.

As indicated in Fig. 4, we will focus our analysis on two of the factors that affect the kinetics of HMs and HACs, $k_1e_2$ and $k_7h_0$, respectively. These quantities regulate the rate at which HMs and HACs form complexes with their corresponding substrates. These processes depend on the availability of cofactors that act as donors of the methyl and acetyl groups. The abundance of such cofactors, S-Adenosyl methionine (SAM) and Acetyl-coA (AcoA), respectively, modulate the rate of the corresponding chromatin modifier, and it therefore provides a natural (and biologically relevant) way of changing these parameters. To tackle this, we have modified the microscopic model and derived the corresponding CG model to account for the effects of the metabolic cofactors. The details of the derivation are given in the Supplementary Materials, Section III.

**Fig. 7:**
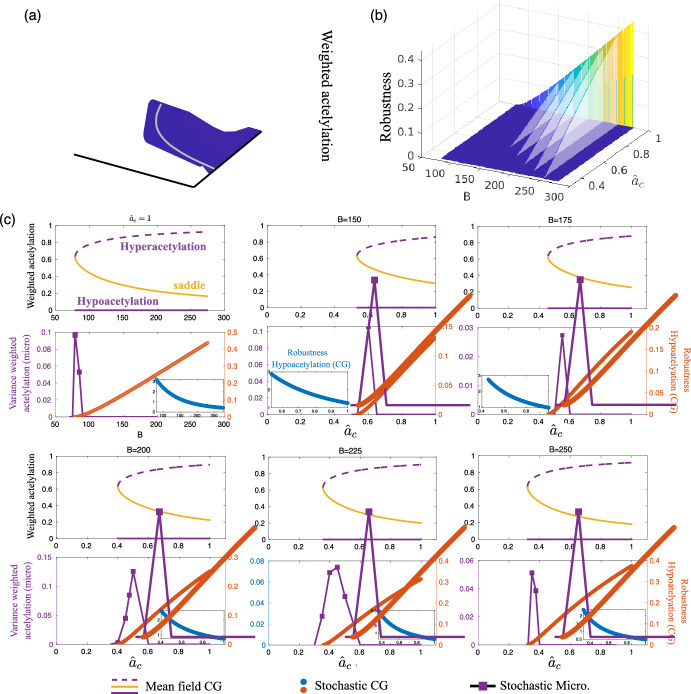
The coarse-grained (CG) approximation accurately predicts the critical behavior of the microscopic system. (a-b) CG analysis (mean-field limit and stochastic diffusion limit, respectively). These analyses in terms of the collective observables (specifically, weighted acetylation) show that competition for chromatin modifiers (HDACs in this specific case) induces a tipping point behavior (c). The analysis of CG stochastic model allows us to characterise the robustness of the stable ELs. When approaching the tipping point, the robustness of the EL becomes vanishingly small. The microscopic system exhibits a tipping point at a critical value of the control parameter that is accurately predicted by the CG approximation, as shown in (c), where the variance of the microscopic system (see purple lines) is observed to acutely increase in the vicinity of the critical point predicted by the CG approximation (orange circles). The sensitivity analysis of the CG robustness predicts that reduced levels of AcoA can facilitate the tipping point, as lowering AcoA levels decreases the robustness of the EL (b). This prediction is also faithfully fulfilled by the microscopic system, as shown in (d)-(h). This analysis has been performed with the all-to-all connectivity matrix. Parameter values are given in Supplementary Materials, Section IV.

By inspection, the CG equations for the HM- and HAC-cofactor-regulated model can be obtained from Eqs. (21)-(22) by making the substitutions $k_1\rightarrow k_1\hat{s}_c$ and $k_7\rightarrow k_7\hat{a}_c$, where the *effective cofactors* $\hat{a}_c$ and $\hat{s}_c$ are

$$\begin{aligned} \hat{a}_c=\frac{K_A{\mathcal A}_T}{\frac{|\textsf{u}|_1}{\sqrt{N_c}}+K_A{\mathcal A}_T}\, \phantom {m} \text{ and }\,\phantom {m} \hat{s}_c=\frac{K_S{\mathcal S}_T}{\frac{|\textsf{u}|_1}{\sqrt{N_c}}+K_S{\mathcal S}_T}, \end{aligned}$$

respectively. Constants $K_A$ and $K_S$ are the affinities and ${\mathcal A}_T$ and ${\mathcal S}_T$ are the levels of AcoA and SAM, respectively. The CG model is the limit of the model modulated by abundance cofactor when the cofactor level tends to infinity (see Supplementary Materials, Section III). Similarly, the HM- and HAC-cofactor-regulated model can be obtained by making the same substitutions in Eqs. (B2)-(B2) in Appendix B. The results of our analysis varying the effective cofactors are shown in Fig. 6.

Studying first the behavior of the CG system, as predicted by our sensitivity analysis, the variation of the effective cofactors affects the robustness of the hyper- and hypo-acetylated states. Focusing on the mean-field limit of the CG dynamics, Fig. 6 shows that reduction of the AcoA effective cofactor, $\hat{a}_c$, induces a saddle-node bifurcation that catastrophically destroys the hyperacetylated state. Results regarding the stochastic system are consistent with the deterministic ones. Figure 6 also shows that the robustness associated with the hyperacetylated state decreases until vanishingly small values are reached, heralding the tipping point where the hyperacetylated state disappears. It is worth noting that the critical value of the stochastic tipping point is equal to the mean-field bifurcation critical value of $\hat{a}_c$. By contrast, as predicted by our sensitivity analysis (see Fig. 4), reduction of the SAM effective cofactor increases the robustness of the hyperacetylated state.

More importantly, our CG analysis predicts the critical behavior of the full microscopic stochastic system. Figure 6 shows simulation results for the microscopic system as the AcoA effective cofactor decreases. Specifically, we have plotted the variance over several sample paths. We can see that as the value of the cofactor approaches the critical value at which the tipping point occurs, i.e., where the robustness of the hyperacetylated state becomes vanishingly small, the variance experiences a very pronounced peak. This behavior (vanishingly small barriers and a peak in the variance) is exactly what one expects at a tipping point (Carpenter and Brock 2006). Beyond that, loss of AcoA interacts synergistically with competition for HACs to induce a tipping point in the system. Since the levels of AcoA are (experimentally) observable, they are candidates to act as MWS for epigenetic tipping points.

## Discussion

Epigenetic landscapes (EL) stability is one of the most important factors in maintaining gene expression patterns in differentiated cells, thereby preserving their specific identities and functions. Recently, competition for chromatin modifiers between different epigenomic substrates (e.g., DSBs vs. histone modification-regulated gene expression) has been proposed as a key factor that can destabilize and cause loss of epigenetic information from EL (Al-Radhawi et al. 2022; Yang et al. 2023; Stepanova et al. 2025b). Here, we developed a first-in-class general framework to analyze tipping points in large ELs. By measuring the sensitivity and robustness of such epigenetic tipping points, we reveal that the availability of metabolic cofactors can operate as a *bona fide* MWS able to anticipate critical transitions in ELs.

We first formulated a theoretical framework and applied it to a relatively simplified epigenetic regulatory system, where a dynamic change between trimethylation and acetylation at H3K27 underlies the switching between repressive and active genomic regions (Newar et al. 2022). To analyze the stability of large-scale EL patterns, our framework includes a pipeline where a coarse-grained (CG) description of the system in terms of a low-dimensional system (two-dimensional, in the case of the system studied here) collective observables was obtained using the spectral reduction dimension (Laurence et al. 2019; Vegué et al. 2023). Subsequently, bifurcation theory has been used to study the CG system in the mean-field limit [Fig. 7(a)] while asymptotic techniques, in particular the WKB approximation, were used to study the corresponding Fokker-Plank equation in the stochastic diffusion limit. This analysis enabled the definition of robustness measures for stable ELs in the multi-stable regime [Fig. 7(b)]. The prediction that the large-scale transitions in ELs associated with chromatin modifier competition occur through tipping points was consistent in both the deterministic and stochastic analyses. These predictions were tested against the benchmark of the microscopic model and were found to accurately reproduce the behavior of the model [see Fig. 7(c)-(h)].

By performing a parameter sensitivity analysis for the robustness metric, we were able to extract more information from the analysis of the stochastic CG approximation. This analysis informed us about the parameters whose variation has a stronger impact on the robustness of a stable EL. In other words, we can determine which parameters are more likely to facilitate the occurrence of a tipping point. Conversely, we can also determine which parameters increase the robustness of the EL and thus prevent the occurrence of the tipping point (see Fig. 4). Importantly, the robustness sensitivity analysis enables us to articulate a biologically feasible mechanism involving SAM and acetyl-CoA, the universal donors that provide methyl and acetyl units for histone modification, to control the robustness of specific stable ELs within the bistable regime (Dodd et al. 2007; Butz et al. 2022). The predictions of the CG approximation again closely and accurately matched the behavior of the microscopic benchmark when we modified the microscopic model to explicitly account for the levels of SAM and acetyl-CoA and derived the corresponding CG approximation (Figs. 6 and 7).

Our framework, which predicts that acetyl-CoA and SAM are key regulators of the robustness of specific ELs, supports the hypothesis that the levels of certain metabo-epigenetic cofactors can be used as EWS to anticipate epigenetic tipping points driven by competition for chromatin modifiers. For example, since reduced levels of acetyl-CoA indicate reduced robustness of the hyperacetylated EL, the status of acetyl-CoA may help to predict the tipping point for initiation of an EL transition (that is, the lower the acetyl-CoA levels, the closer the tipping point). The abundance and/or distribution of metabolo-epigenetic cofactors changes significantly in response to several physiological or pathological conditions in extracellular and subcellular reservoirs (Etchegaray and Mostoslavsky 2016; Campbell and Wellen 2018; Sivanand et al. 2018; Ye and Tu 2018; Haws et al. 2020a, b; Izzo et al. 2021). Acetyl-CoA levels rise and fall with nutrient abundance, oncogenic signaling, and microenvironmental conditions, and such changes in acetyl-CoA abundance can regulate histone acetylation at specific loci and the expression of distinct sets of genes. Acetyl-CoA status can also be coordinated also with chromatin structure and function through spatiotemporal control of nuclear acetyl-CoA concentration via post-translational modification and/or dynamic chromatin recruitment and localization of acetyl-CoA-producing enzymes  (Etchegaray and Mostoslavsky 2016; Campbell and Wellen 2018; Sivanand et al. 2018; Ye and Tu 2018; Haws et al. 2020a, b; Izzo et al. 2021). Serine starvation, a common microenvironmental stress in breast cancer that leads to a marked and rapid insufficiency of the nucleocytosolic reservoir of acetyl-CoA, has recently been shown to deplete H3K27 acetylation and induce a global transition to an hypoacetylated EL (Li et al. 2023). Given that the acute depletion of acetyl-CoA culminates in epigenetic reprogramming and silencing of a plethora of genes important for defining cell lineage commitment, such as those involved in the cellular response to estrogen (Li et al. 2023), these results experimentally support our proposal that, in response to small changes in the environmental availability of metabolic cofactors (e.g., acetyl-CoA) near a tipping point, critical transitions may occur driven by competition for chromatin modifiers. Accordingly, the provision of acetate, an alternate source of acetyl-CoA, is sufficient to restore EL and prevent estrogen receptor (ER) silencing under serine-free conditions (Li et al. 2023). In analogy to acetyl-CoA, systemic and local changes in SAM-producing pathways may also contribute to the variability of histone methylation patterns and chromatin regulation of different functional cellular states in tissues and organs. The dietary and extracellular availability of methionine and serine significantly affect SAM levels and histone methylation, and we are beginning to realize that nuclear SAM production may also be important for the function of specific chromatin-associated complexes that regulate histone methylation (Etchegaray and Mostoslavsky 2016; Campbell and Wellen 2018; Sivanand et al. 2018; Ye and Tu 2018; Haws et al. 2020a, b; Izzo et al. 2021). The growing evidence that acetyl-CoA and SAM are key messengers that translate nutrient-dependent and compartment-specific metabolic signals into chromatin modifications not only provides a robust biological basis for our proposal of metabolic cofactors as EWS that can predict the onset of EL global transitions but also suggests metabolic strategies (e.g., dietary interventions, targeted modification of metabolic pathways) to avoid them.

Our sensitivity analysis shows that in addition to metabolic (co)factors affecting the activity of chromatin modifiers, the structure and intensity of the interactions are also factors that quantifiably affect the robustness of bistable EL (summarized within the parameter $\hat{\Phi }$, as shown in Fig. 4). To address this issue in more detail, we considered three connectivity patterns, namely all-to-all, heterogeneous, and first-neighbours considering also a particular case with ablation. In addition to demonstrating that our CG method can handle a wide variety of interaction patterns and accurately predict the critical behavior of the system (Eqs. (B2)-(B3)), an ablation study shows that our model does not require long-range interactions to generate bistable chromatin. Interactions in the range of $\sim$5 nucleosomes ($\sim$750 base pairs) are sufficient to confer such a property. Our model uniquely approaches the activity of chromatin modifiers using enzyme kinetics rather than (recruited, nonlinear) binding/unbinding kinetics (Dodd et al. 2007; Sneppen and Dodd 2016; Jost and Vaillant 2018; Sneppen and Ringrose 2019). The nonlinearity that this feature introduces into the kinetics of chromatin modifiers, facilitates the robust production of bistable behavior. In line with the results of previous models (Dodd et al. 2007; Sneppen and Dodd 2016; Jost and Vaillant 2018; Sneppen and Ringrose 2019), the introduction of longer-range interactions (even weak ones) helps to increase the robustness of bistable chromatin.

The scope of the present study is somehow limited by the steady-state analysis we have performed. If cells are undergoing the cell cycle, the histone modification levels are, on average, halved upon DNA replication. Modifications accumulate again during the remainder of the cell cycle. This phenomenon is not accounted for in the current analysis. Histone modification halving is a major perturbation that is likely to affect the tipping points of the system. Although this limits the applicability of the current study, our analysis still has interest in situations such as malignant transformations in, e.g., epithelial cells. These are fully differentiated cells that have exited the cell cycle. However, loss of epigenetic information can induce loss of cell identity, leading to malignant transformation.

Finally, there are several ways to extend our current framework to account for tipping points in multistable ELs. One obvious extension would be to consider more chromatin modifiers that generate more complex EL, particularly those that involve rugged, non-uniform patterns of chromatin modifications (Yang et al. 2023) This would allow computational testing of recently proposed models of epigenetic information storage in which histone modifications such as H3K27me3 become dysregulated over time (Kane and Sinclair 2019; Yang et al. 2023; Lu et al. 2023).

As EL transitions are beginning to emerge as driving mechanisms for critical transitions in epithelial-hybrid-mesenchymal cell fate determination that accompany metastatic, drug-resistance phenomena during cancer progression (Sarkar et al. 2019; Deb et al. 2022)) and disruption of developmental genes during aging (Kane and Sinclair 2019; Yang et al. 2023; Lu et al. 2023; Cipriano et al. 2024), our mathematical methodology opens a new avenue for obtaining improved EWS of more complex epigenetic tipping points for both theoretical and practical purposes. Importantly, the ability to anticipate critical EL transitions using the abundance of certain metabolic cofactors as EWS may provide predictive biomarkers and uncover new opportunities for therapeutic restriction of pathological cell fate decisions, but also advance the application of therapies to restore cellular identity in post-injury regeneration. In this regard, exogenous supplementation of a few metabolites, including methionine and SAM, that mimic an endogenous one-carbon metabolomic wave that occurs during early cell transitions in differentiation has been shown to increase global acetylation marks and prime cell identity changes to promote faster regeneration after muscle injury in young and aged mice (Hernandez-Benitez et al. 2024). These findings strongly suggest that our current proposal of epigenetic metabolites as EWS for critical EL transitions may provide a translatable strategy to metabolically redirect cell identity and restore cell and tissue function during disease and aging.

## Supplementary Information

Below is the link to the electronic supplementary material.

**Supplementary information:** A supplementary materials and methods file accompanies this manuscript, which also includes supplementary figures and results. (pdf 1,708KB)

## Stochastic Microscopic Model: Reaction Rates, Scaling, and Non-dimensionalisation

***Reaction rates*** The microscopic model is implemented using Law-of-Mass Action rates (Ingalls 2013). Each of the three reactions involved in the enzyme-activated (de)methylation and (de)acetylation of the H3K27 residues within each of the sub-regions considered is modelled in this way. The resulting reaction rates are given in Table 2.

**Table 2:** Table showing the reaction rates associated with the model of chromatin modifications with S-Adenosyl methionine and acetyl-coA cofactors. All the entries $r_{ijk}=0$ except for those shown in the table below. $i=1,\dots ,N_s$, $j=1,\dots ,N_C$, and $k=1,\dots ,R$. The number of species, regions, and reactions are denoted by $N_s=18$, $N_c$, and $R=16$, respectively. We only show the non-zero stoichiometric coefficients.

**Modification**	**Rate**	**LMA rate**	**Stoichiometric coeff.**		
Methylation	$W_{(j-1)R+1}$	$k_{1_j}S_jE_2$	$r_{1j1}=-1$	$r_{4j1}=-1$	$r_{5j1}=+1$
	$W_{(j-1)R+2}$	$k_{2_j}C_{E_j}$	$r_{1j2}=+1$	$r_{4j2}=+1$	$r_{5j2}=-1$
	$W_{(j-1)R+3}$	$k_{3_j}C_{E_j}$	$r_{2j3}=+1$	$r_{5j3}=-1$	$r_{2j3}=+1$
Demethylation	$W_{(j-1)R+4}$	$k_{4_j}M_jE_U$	$r_{2j4}=-1$	$r_{6j4}=-1$	$r_{7j4}=+1$
	$W_{(j-1)R+5}$	$k_{5_j}C_{U_j}$	$r_{2j5}=+1$	$r_{6j5}=+1$	$r_{7j7}=-1$
	$W_{(j-1)R+6}$	$k_{6_j}C_{U_j}$	$r_{6j6}=+1$	$r_{7j6}=-1$	$r_{1j6}=+1$
Acetylation	$W_{(j-1)R+7}$	$k_{7_j}S_jE_{HA}$	$r_{1j7}=-1$	$r_{8j7}=-1$	$r_{9j7}=+1$
	$W_{(j-1)R+8}$	$k_{8_j}C_{HA_j}$	$r_{1j8}=+1$	$r_{8j8}=+1$	$r_{9j8}=-1$
	$W_{(j-1)R+9}$	$k_{9_j}C_{HA_j}$	$r_{8j9}=+1$	$r_{9j9}=-1$	$r_{3j9}=+1$
Deacetylation	$W_{(j-1)R+10}$	$k_{10_j}A_jE_S$	$r_{3j10}=-1$	$r_{10j10}=-1$	$r_{11j10}=+1$
	$W_{(j-1)R+11}$	$k_{11_j}C_{S_j}$	$r_{3j11}=+1$	$r_{10j11}=+1$	$r_{11j11}=-1$
	$W_{(j-1)R+12}$	$k_{12_j}C_{S_j}$	$r_{10j12}=+1$	$r_{11j12}=-1$	$r_{1j12}=+1$
Sirtuin sequestration	$W_{(j-1)R+13}$	$k_{13_j}DE_S$	$r_{10j13}=-1$	$r_{12j13}=+1$	
	$W_{(j-1)R+14}$	$k_{14_j}C_{S_D}$	$r_{10j14}=+1$	$r_{12j14}=-1$	
Cofactor binding	$W_{(j-1)R+15}$	$k_{15_j}AE_H$	$r_{13j15}=-1$	$r_{8j15}=+1$	
(AcoA)	$W_{(j-1)R+16}$	$k_{16_j}E_{HA}$	$r_{13j16}=+1$	$r_{8j16}=-1$	
Cofactor binding	$W_{(j-1)R+17}$	$k_{17_j}SE_2$	$r_{13j17}=-1$	$r_{8j17}=+1$	
(SAM)	$W_{(j-1)R+18}$	$k_{18_j}E_{2S}$	$r_{13j18}=+1$	$r_{8j18}=-1$	

***Conservation laws*** The above system of enzymatic reaction has $N_C$ local conservation laws, expressing the conservation of the number of modification residues within each of the $N_C$ chromatin sub-regions:

$$\begin{aligned} S_i+M_i+A_i+C_{E_i}+C_{U_i}+C_{H_i}+C_{S_i}=S_{27_i},\,i=1,\dots ,N_C, \end{aligned}$$

and 4 global conservation laws, corresponding to the conservation of each chromatin-modifying enzyme:

$$\begin{aligned} E_2+\sum _iC_{E_i}=E_0, E_U+\sum _iC_{U_i}=U_0, E_H+\sum _iC_{H_i}=H_0, E_S+C_{S_D}+\sum _iC_{S_i}=S_0, \end{aligned}$$

***Scaling***

The multiscale nature of epigenetic gene regulatory netowkrs has been shown to be an essential factor of the dynamics of such systems (Folguera-Blasco et al. 2018, 2019; Alarcón et al. 2021). In a nutshell, the disparity between the characteristic scales of the different actors involved in the model induces separation of time scales whereby the chemical species whose characteristic abundances are smaller (typically, enzymes) evolve much faster than their more abundant counterparts (unmodified chromatin residues) (Keener and Sneyd 1998; Ingalls 2013; Kang et al. 2014).

Consider the quantities $\Omega _{27}$ and and $\Omega _E$. These quantities are such that:

$$\begin{aligned} \textbf{s}=\Omega _{27}^{-1} \textbf{S},\;\textbf{a}=\Omega _{27}^{-1} \textbf{A},\;\textbf{m}=\Omega _{27}^{-1} \textbf{M}, \end{aligned}$$

$$\textbf{s}=\{s_i\}_{i=1,\dots ,N_c}$$, $$\textbf{S}=\{S_i\}_{i=1,\dots ,N_c}$$, $$\textbf{a}=\{a_{i}\}_{i=1,\dots ,N_c}$$, $$\textbf{A}=\{A_{i}\}_{i=1,\dots ,N_c}$$, $$\textbf{m}=\{m_{i}\}_{i=1,\dots ,N_c}$$, and $$\textbf{M}=\{M_{i}\}_{i=1,\dots ,N_c}$$;

$$\begin{aligned} \mathbf{c_J}=\Omega _E^{-1} \mathbf{C_J},\;\mathbf{e_J}=\Omega _E^{-1} \mathbf{E_J}, \end{aligned}$$

$\mathbf{c_J}=\{c_{J_i}\}_{i=1,\dots ,N_c}$, $\mathbf{C_J}=\{C_{J_i}\}_{i=1,\dots ,N_c}$, $\mathbf{e_J}=\{e_{J_i}\}_{i=1,\dots ,N_c}$, and $\mathbf{E_J}=\{E_{J_i}\}_{i=1,\dots ,N_c}$, for $J=2,U,H,S$. The characteristic scales are assumed to satisfy (Folguera-Blasco et al. 2018, 2019) $\Omega _{27}\gg \Omega _E$.

***Non-dimensionalisation*** Furthermore, we will rescale the law-of-mass-action rates, $W_i$ as:

A1 $$\begin{aligned} W_i(\textsf{X})=k_2\Omega _E\Omega _{27}^2w_i(\textsf{x})+{\mathcal O}(\Omega _{27}^0,\Omega _E^0) \end{aligned}$$

**Table 3:** Dimensionless parameters.

**Parameter**	**Dimensionless parameter**
$k_{1_j}$	$\hat{k}_{1_j}=\frac{k_{1_j}}{k_{2_j}\Omega _{27}}$
$k_{2_j}$	$\hat{k}_{2_j}=\frac{k_{2_j}}{\Omega _{27}^2}$
$k_{3_j}$	$\hat{k}_{3_j}=\frac{k_{3_j}}{k_{2_j}\Omega _{27}^2}$
$k_{4_j}$	$\hat{k}_{4_j}=\frac{k_{4_j}}{k_{2_j}\Omega _{27}}$
$k_{5_j}$	$\hat{k}_{5_j}=\frac{k_{5_j}}{k_{2_j}\Omega _{27}^2}$
$k_{6_j}$	$\hat{k}_{6_j}=\frac{k_{6_j}}{k_{2_j}\Omega _{27}^2}$
$k_{7_j}$	$\hat{k}_{7_j}=\frac{k_{7_j}}{k_{2_j}\Omega _{27}}$
$k_{8_j}$	$\hat{k}_{8_j}=\frac{k_{8_j}}{k_{2_j}\Omega _{27}^2}$
$k_{9_j}$	$\hat{k}_{9_j}=\frac{k_{9_j}}{k_{2_j}\Omega _{27}^2}$
$k_{10_j}$	$\hat{k}_{10_j}=\frac{k_{10_j}}{k_{2_j}\Omega _{27}}$
$k_{11_j}$	$\hat{k}_{11_j}=\frac{k_{11_j}}{k_{2_j}\Omega _{27}^2}$
$k_{12_j}$	$\hat{k}_{12_j}=\frac{k_{12_j}}{k_{2_j}\Omega _{27}^2}$
$k_{13_j}$	$\hat{k}_{13_j}=\frac{k_{13_j}}{k_{2_j}\Omega _{27}^2}$
$k_{14_j}$	$\hat{k}_{14_j}=\frac{k_{14_j}}{k_{2_j}\Omega _{27}^2}$
$k_{15_j}$	$\hat{k}_{15_j}=\frac{k_{15_j}}{k_{2_j}\Omega _{27}^2}$
$k_{16_j}$	$\hat{k}_{16_j}=\frac{k_{16_j}}{k_{2_j}\Omega _{27}^2}$
$k_{17_j}$	$\hat{k}_{17_j}=\frac{k_{17_j}}{k_{2_j}\Omega _{27}^2}$
$k_{18_j}$	$\hat{k}_{18_j}=\frac{k_{18_j}}{k_{2_j}\Omega _{27}^2}$

where $\textsf{X}=(\textbf{S}^T,\textbf{A}^T,\textbf{M}^T,\{\textbf{E}^T_{J},\textsf{C}^T_J\}_{J=2,U,H,S})^T$ and $\textsf{x}=(\textbf{s}^T,\textbf{a}^T,\textbf{m}^T,\{\textsf{e}^T_{J},\textsf{c}^T_J\}_{J=2,U,H,S})^T$, respectively. Similarly, we re-scale time using: $\hat{t}=k_2\Omega _E\Omega _{27}t$. The rationale behind this choice of scaling is fully discussed in Alarcón (2014); de la Cruz et al. (2015). This rescaling implies a non-dimensionalisation of the model kinetic parameters that is shown in Table 3

## Mean-field Equations of the Microscopic System

Assuming, as per the scaling assumption in Section Appendix A, the quasi-steady state approximation, the mean-field equations of the microscopic model, Eqs. (5)-(6), Section 2.1, are

B2 $$\begin{aligned} &  \frac{dm_i}{dt}=k_3\frac{k_1e_0\left( \alpha _1+\sum _{j}w_{ij}m_j\right) u_i}{k_2+k_3+k_1\left( \alpha _1+\sum _{j}w_{ij}m_j\right) u_i}- k_6\frac{k_4u_0\left( \alpha _1+\sum _{j}w_{ij}a_j\right) m_i}{k_5+k_6+k_4\left( \alpha _1+\sum _{j}w_{ij}a_j\right) m_i}, \end{aligned}$$

B3 $$\begin{aligned} &  \frac{da_i}{dt}=k_9\frac{k_7h_0\left( \alpha _7+\sum _{j}w_{ij}a_j\right) u_i}{k_8+k_9+k_7\left( \alpha _7+\sum _{j}w_{ij}a_j\right) u_i}- k_{12}\frac{k_{10}e_{s_0}\left( \alpha _{10}+\sum _{j}w_{ij}m_j\right) a_i}{k_{11}+k_{12}+B+k_{10}\left( \alpha _{10}+\sum _{j}w_{ij}m_j\right) a_i},\quad \end{aligned}$$

where $i=1,\dots ,N_C$. Here, $\{m_i\}_{i=1}^{N_{C}}$ and $\{a_i\}_{i=1}^{N_C}$ are the levels of methylation and acetylation in sunregion *i*, and *B* is the QSS rate of sequestration of SIRT by DNA-damaged sites, which is proportional to the amount of DSBs (Stepanova et al. 2025a). For simplicity, we have dropped the ”hat” notation for the dimensionless variables (see Table 3).

## Closure Conditions for the Spectral Dimension Reduction

Here we derive the closure conditions that the parameters $\phi$, $\gamma$, $\{\Phi _i\}_{i=1}^2$, and $\{\Gamma _i\}_{i=1}^2$ must satisfy in order to the first order corrections to the SDR coarse-grained description vanish.

Consider first the functions $f_i$ and $F_{i,k}$ and their Taylor expansions around $\textsf{x}_j=\phi {\mathcal X}$, and $x_{ij}=\Phi _1{\mathcal X}_{i}$ and $x_{kl}=\Phi _2{\mathcal X}_k$, respectively:

$$\begin{aligned} \sum _ju_jf_i(\textsf{x}_j)=f_i(\phi {\mathcal X})\sum _ju_j+\sum _kf_{i,k}(\phi {\mathcal X})\sum _ju_j(x_{kj}-\phi {\mathcal X}_k)+{\mathcal O}(\Delta ^2) \end{aligned}$$

$$\begin{aligned}&\sum _ju_jw_{jl}F_{i,k}(x_{ij},x_{kl})\\&\quad =F_{i,k}(\Phi _1{\mathcal X}_{i},\Phi _2{\mathcal X}_k)\sum _ju_jw_{jl}+F_{i,k,1}(\Phi _1{\mathcal X}_i,\Phi _2{\mathcal X}_k)\sum _{j,l}u_jw_{jl}(x_{ij}-\Phi _1{\mathcal X}_i)\\&\qquad +F_{i,k,2}(\Phi _1{\mathcal X}_i,\Phi _2{\mathcal X}_k)\sum _{j,l}u_jw_{jl}(x_{kl}-\Phi _2{\mathcal X}_k)+{\mathcal O}(\Delta ^2)\end{aligned}$$

For the first-order corrections in these Taylor expansions to identically vanish, the following conditions must be satisfied (Laurence et al. 2019):

C4 $$\begin{aligned} &  1-|u|_1\phi =0, \end{aligned}$$

C5 $$\begin{aligned} &  \textsf{x}_k^T\cdot \textsf{W}^T\cdot \textsf{u}-\alpha _w\Phi _2\textsf{x}_k^T\cdot \textsf{u}=0\Rightarrow \textsf{W}^T\cdot \textsf{u}=\alpha _w\Phi _2\textsf{u},\end{aligned}$$

C6 $$\begin{aligned} &  \textsf{x}_k^T\cdot \textsf{K}\cdot \textsf{u}-\alpha _w\Phi _1\textsf{x}_k^T\cdot \textsf{u}=0\Rightarrow \textsf{K}\cdot \textsf{u}=\alpha _w\Phi _1\textsf{u}, \end{aligned}$$

where $|u|_1$ is L1-norm of $\textsf{u}$, $\alpha _w=\sum _{ij}u_jw_{ij}$, and

$$\begin{aligned} \textsf{K}=(k_{ij})=\left\{ \begin{array}{l}k_{ii}=\sum _jw_{ij} \text{ if } i=j,\\ k_{ij}=0 \text{ otherwise }.\end{array}\right. \end{aligned}$$

As pointed out in Laurence et al. (2019), conditions (C5) and (C6) cannot (in general) be satisfied simultaneously. To circumvent this, the solution is proposed whereby condition (C5) is satisfied exactly, whereas condition (C6) is satisfied only approximately, in a variational sense. Following this prescription, we assume that $\textsf{u}$ is the dominant eigenvector of $\textsf{W}^T$ with eigenvalue $\lambda _D$ (as per condition (C5)). This implies that $\alpha _w\Phi _2=\lambda _D$, so that $\Phi _2=|\textsf{u}|_1^{-1}$. As for condition (C6), $\Phi _1$ is determined in a variational way by requiring that $\Phi _1^*=\min _{\Phi _1}M(\Phi _1)$ where

$$\begin{aligned} M_F(\Phi _1)=\Vert \textsf{K}\cdot \textsf{u}-\alpha _w\Phi _1\textsf{u}\Vert ^2 \end{aligned}$$

so that

$$\begin{aligned} \Phi _1^*=\frac{\textsf{u}^T\cdot \textsf{K}\cdot \textsf{u}}{\alpha _w\textsf{u}^T\cdot \textsf{u}} \end{aligned}$$

Finally, the drift term of Eqs. (7) and (8) is given by:

C7 $$\begin{aligned} \sum _{j,k}u_jr_{ijk}\omega _{(j-1)R+k}(\textsf{x})=|u|_1f_i\left( \frac{{\mathcal X}}{|u|_1}\right) +\alpha _w\sum _kF_{i,k}\left( \Phi _1^*{\mathcal X}_i,\frac{{\mathcal X}_k}{|\textsf{u}|_1}\right) +{\mathcal O}(\Delta ^2) \end{aligned}$$

Regarding the noise term, we proceed in a similar way by performing the Taylor expansion of the functions $g_i$ and $G_{i,k}$ around $\textsf{x}_j=\gamma {\mathcal X}$, and $x_{ij}=\Gamma _{1,j}{\mathcal X}_{i}$ and $x_{kl}=\Gamma _{2,j}{\mathcal X}_k$, respectively:

$$\begin{aligned} \sum _ju^2_jg_i(\textsf{x}_j)=g_i(\gamma {\mathcal X})\sum _ju^2_j+\sum _kg_{i,k}(\gamma {\mathcal X})\sum _ju^2_j(x_{kj}-\gamma {\mathcal X}_k)+{\mathcal O}(\Delta ^2) \end{aligned}$$

$$\begin{aligned}&\sum _{j,l}u^2_jw_{jl}G_{i,k}(x_{ij},x_{kl})\\&\quad =\sum _{j,l}u^2_jw_{jl}G_{i,k}(\Gamma _{1,i}{\mathcal X}_{i},\Gamma _{2,j}{\mathcal X}_k)+\sum _{j,l}u^2_jw_{jl}G_{i,k,1}(\Gamma _{1,j}{\mathcal X}_i,\Gamma _{2,j}{\mathcal X}_k)(x_{ij}-\Gamma _{1,j}{\mathcal X}_i)\\&\qquad +\sum _{j,l}u^2_jw_{jl}G_{i,k,2}(\Gamma _{1,j}{\mathcal X}_i,\Gamma _{2,j}{\mathcal X}_k)(x_{kl}-\Gamma _{2,j}{\mathcal X}_k)+{\mathcal O}(\Delta ^2)\end{aligned}$$

The first condition that we obtain for the first order contribution to vanish is:

C8 $$\begin{aligned} 1-\gamma \Vert \textsf{u}\Vert ^2=0. \end{aligned}$$

Recall that $G_{i,k}(z,y)\propto zy$ and therefore $G_{i,k,1}(z,y)\propto y$ and $G_{i,k,2}(z,y)\propto z$. In view of this specific form of the functions, $G_{i,k}$ if we choose

$$\begin{aligned} \Gamma _{1,j}=\frac{\Gamma _1}{u_j},\;\Gamma _{2,j}=\Gamma _2 \end{aligned}$$

(where $\Gamma _1$ and $\Gamma _2$ are constants), we obtain an additional constraint which reads

C9 $$\begin{aligned} \textsf{x}_k^T\cdot \textsf{W}^T\cdot \textsf{u}-\alpha _w\Gamma _2\textsf{x}_k^T\cdot \textsf{u}\Rightarrow \textsf{W}^T\cdot \textsf{u}=\alpha _w\Gamma _2\textsf{u} \end{aligned}$$

which is identical to condition (C5) and therefore $\alpha _w\Gamma _2=\lambda _D$, so that ${\Gamma _2}=|\textsf{u}|_1^{-1}$.

The third and final condition that needs to be satisfied is given by

$$\begin{aligned} \textsf{x}_k^T\cdot \textsf{K}\cdot \textsf{v}=\alpha _w\Gamma _1\textsf{x}_k^T\cdot \textsf{u}. \end{aligned}$$

The vector $\textsf{v}=(u_i^2)_{i=1}^{N_C}$. As in the case of the drift term, we tackle this condition variationally, i.e., $\Gamma _1^*=\min _{\Gamma _1}M_G(\Gamma _1)$ with

$$\begin{aligned} M_G=\Vert \textsf{K}\cdot \textsf{v}-\alpha _w\Gamma _1\textsf{u}\Vert ^2 \end{aligned}$$

so that

C10 $$\begin{aligned} \Gamma _1^*=\frac{\textsf{u}^T\cdot \textsf{K}\cdot \textsf{v}}{\alpha _w\textsf{u}^T\cdot \textsf{u}}. \end{aligned}$$

Finally, taking into account that $G_{i,k}\left( \frac{\Gamma _1^*}{u_j}{\mathcal X}_i,\frac{{\mathcal X}_k}{|\textsf{u}|_1}\right) \sim \frac{\Gamma _1^*}{u_j}{\mathcal X}_i\frac{{\mathcal X}_k}{|\textsf{u}|_1}$the noise term is given by

C11 $$\begin{aligned} \sum _{j,k}u^2_jr^2_{ijk}\omega _{(j-1)R+k}(\textsf{x})=\Vert u\Vert ^2g_i\left( \frac{{\mathcal X}}{\Vert u\Vert ^2}\right) +\alpha _w\sum _kG_{i,k}\left( \Gamma _1^*{\mathcal X}_i,\frac{{\mathcal X}_k}{|\textsf{u}|_1}\right) +{\mathcal O}(\Delta ^2) \end{aligned}$$

***Dealing with the conservation laws***

The global conservation law regarding the total concentration of enzymes is a roadblock in the spectral reduction method that we need to deal with. Consider, without loss of generality, the concentration of sirtuins, $e_S$. Recall that $e_S$ and the complexes $c_{S_i}$ are constrained by:

$$\begin{aligned} e_S=s_{0}-c_{S_D}-\sum _{i}c_{S_i}, \end{aligned}$$

Consider the former, which we rewrite as $e_S=s_{0}-C_D-\textsf{c}_{S}^T\cdot \textsf{1}$, where $\textsf{1}^T=(1,\dots ,1)$, and $\textsf{1}=\sum _{\alpha }(\textsf{u}_{\alpha }^T\cdot \textsf{1})\textsf{u}_{\alpha }$, where $\{u_{\alpha }\}$ is the base formed by the eigenvectors of $\textsf{W}$ ordered in descending order of their respective eigenvalues (recall that $\textsf{u}\equiv \textsf{u}_1$ and $\lambda _D=\lambda _1$). If $\textsf{u}^T\cdot \textsf{1}\ll \textsf{u}_{\alpha }^T\cdot \textsf{1}$ for all $\alpha > 1$, we can approximate the conservation law by:

$$\begin{aligned} e_S\simeq s_{0}-c_{S_D}-(\textsf{u}^T\cdot \textsf{1})\textsf{c}_{S}^T\cdot \textsf{u}=s_{0}-c_{S_D}-(\textsf{u}^T\cdot \textsf{1}){\mathcal X}_{11}. \end{aligned}$$

where ${\mathcal X}_{11}=\textsf{c}_{S}^T\cdot \textsf{u}$. However, this conservation law is now unbalanced in the following sense. Since, assuming that the eigenvectors $\textsf{u}_{\alpha }$ have been normalized so that $\Vert \textsf{u}_{\alpha }\Vert ^2=1$ for all $\alpha$, $\Vert \textsf{1}\Vert ^2=(\textsf{u}^T\cdot \textsf{1})^2+\sum _{\alpha >1}(\textsf{u}_{\alpha }^T\cdot \textsf{1})^2$, by neglecting the $\alpha >1$ terms, we have removed part of the *mass* of the corresponding term in the conservation equation. To compensate the conservation law, we renormalise the other terms of the conservation equation by factor $\frac{\textsf{u}^T\cdot \textsf{1}}{\sqrt{N_C}}$ which corresponds to the fraction of the mass that we have kept in doing our approximation (recall that $\Vert \textsf{1}\Vert ^2=N_C$), so that finally we have that

C12 $$\begin{aligned} e_S\simeq \textsf{u}^T\cdot \textsf{1}\left( \frac{1}{\sqrt{N_c}}s_{0}-\frac{1}{\sqrt{N_c}}c_{S_D}-{\mathcal X}_{11}\right) . \end{aligned}$$

Similarly, the renormalized conservation equations for $e_2$, $e_U$, and $e_H$ read now:

C13 $$\begin{aligned} &  e_2\simeq \textsf{u}^T\cdot \textsf{1}\left( \frac{1}{\sqrt{N_c}}e_{0}-{\mathcal X}_5\right) \end{aligned}$$

C14 $$\begin{aligned} &  e_U\simeq \textsf{u}^T\cdot \textsf{1}\left( \frac{1}{\sqrt{N_c}}u_{0}-{\mathcal X}_{7}\right) \end{aligned}$$

C15 $$\begin{aligned} &  e_H\simeq \textsf{u}^T\cdot \textsf{1}\left( \frac{1}{\sqrt{N_c}}h_{0}-{\mathcal X}_{9}\right) \end{aligned}$$

where $${\mathcal X}_{5}=\textsf{c}_{2}^T\cdot \textsf{u}$$, $${\mathcal X}_{7}=\textsf{c}_{U}^T\cdot \textsf{u}$$, and $${\mathcal X}_{9}=\textsf{c}_{H}^T\cdot \textsf{u}$$.

## Singular Perturbation Analysis of the Multi-scale Coarse-grained Stochastic Dynamical System: Additional Formulae

Taking into account the conservation laws, the system can be reduced to two slow variables ${\mathcal X}_s\equiv ({\mathcal X}_1,{\mathcal X}_2)$ (corresponding to the collective methylation and acetylation, respectively) and four fast variables ${\mathcal X}_f\equiv \{{\mathcal X}_i\}_{i=5,7,9,11}$ (associated with the collective complexes). The drift and noise terms associated with the latter and Eq. (15) are

1. EZH2-unmodified chromatin complex (${\mathcal C}_2$), ${\mathcal X}_5$
  - $${\mathcal D}_5=k_{1}\alpha _1e_2{\mathcal X}_3-(k_2+k_3){\mathcal X}_5+\frac{k_1\alpha _w \Phi _1^*}{|\textsf{u}|_1}e_2{\mathcal X}_1{\mathcal X}_3$$.
  - $${\mathcal N}_5=k_{1}\alpha _1e_2{\mathcal X}_3+(k_2+k_3){\mathcal X}_5+\frac{k_1\alpha _w \Gamma _1^*}{|\textsf{u}|_1}e_2{\mathcal X}_1{\mathcal X}_3$$.
2. UTX-methylated chromatin complex (${\mathcal C}_U$), ${\mathcal X}_{7}$
  - $${\mathcal D}_{7}=k_{4}\alpha _{4}e_U{\mathcal X}_1-(k_{5}+k_{6}){\mathcal X}_{7}+\frac{k_{4}\alpha _w \Phi _1^*}{|\textsf{u}|_1}e_U{\mathcal X}_2{\mathcal X}_1$$.
  - $${\mathcal N}_{7}=k_{4}\alpha _{4}e_U{\mathcal X}_1+(k_{5}+k_{6}){\mathcal X}_{7}+\frac{k_{4}\alpha _w \Gamma _1^*}{|\textsf{u}|_1}e_U{\mathcal X}_2{\mathcal X}_1$$.
3. Histone acetylase-unmodified chromatin complex (${\mathcal C}_H$), ${\mathcal X}_9$
  - $${\mathcal D}_9=k_{7}\alpha _7e_H{\mathcal X}_3-(k_8+k_9){\mathcal X}_9+\frac{k_7\alpha _w \Phi _1^*}{|\textsf{u}|_1}e_H{\mathcal X}_2{\mathcal X}_3$$.
  - $${\mathcal N}_9=k_{7}\alpha _7e_H{\mathcal X}_3+(k_8+k_9){\mathcal X}_9+\frac{k_7\alpha _w \Gamma _1^*}{|\textsf{u}|_1}e_H{\mathcal X}_2{\mathcal X}_3$$.
4. Histone deacetylase (sirtuin)-acetylated chromatin complex (${\mathcal C}_S$), ${\mathcal X}_{11}$
  - $${\mathcal D}_{11}=k_{10}\alpha _{10}e_S{\mathcal X}_2-(k_{11}+k_{12}){\mathcal X}_{11}+\frac{k_{10}\alpha _w \Phi _1^*}{|\textsf{u}|_1}e_S{\mathcal X}_1{\mathcal X}_2$$.
  - $${\mathcal N}_{11}=k_{10}\alpha _{10}e_S{\mathcal X}_2+(k_{11}+k_{12}){\mathcal X}_{11}+\frac{k_{10}\alpha _w \Gamma _1^*}{|\textsf{u}|_1}e_S{\mathcal X}_1{\mathcal X}_2$$.

where ${\mathcal X}_3=|u|_1{\mathcal S}_0-{\mathcal X}_1-{\mathcal X}_2$ and $e_2$, $e_U$, $e_H$, and $e_S$ are given by Eqs. (C12)-(C15).

The analytical expression for the averaged drift and noise terms associated with the slow collective variables ${\mathcal X}_1$ (methylation) and ${\mathcal X}_2$ (acetylation) in Eq. (16) are given by

D16 $$\begin{aligned} &  \langle {\mathcal D}_1\rangle =k_3{\mathcal X}^*_5-k_6{\mathcal X}^*_7\nonumber \\ &  \langle {\mathcal N}_1\rangle =k_{1}e_2\left( \alpha _1+\frac{\alpha _w \Gamma _1^*}{|\textsf{u}|_1}{\mathcal X}_1\right) {\mathcal X}_3+k_2{\mathcal X}^*_5+k_{4}e_U\left( \alpha _4+\frac{\alpha _w \Gamma _1^*}{|\textsf{u}|_1}{\mathcal X}_2\right) {\mathcal X}_1+k_5{\mathcal X}^*_7\nonumber \\ &  \hspace{1.0cm}=k_1\left( \left( \frac{2k_2+k_3}{k_2+k_3}\right) \alpha _1+\frac{\alpha _w}{|\textsf{u}|_1}\left( \Gamma _1^*+\frac{k_2}{k_2+k_3}\Phi _1^*\right) {\mathcal X}_1\right) e_2{\mathcal X}_3+\end{aligned}$$

D17 $$\begin{aligned} &  \hspace{1.5cm} k_4\left( \left( \frac{2k_5+k_6}{k_5+k_6}\right) \alpha _4+\frac{\alpha _w}{|\textsf{u}|_1}\left( \Gamma _1^*+\frac{k_5}{k_5+k_6}\Phi _1^*\right) {\mathcal X}_2\right) e_U{\mathcal X}_1 \end{aligned}$$

and

D18 $$\begin{aligned} &  \langle {\mathcal D}_2\rangle =k_9{\mathcal X}^*_9-k_{12}{\mathcal X}^*_{11}\nonumber \\ &  \langle {\mathcal N}_2\rangle =k_{7}e_H\left( \alpha _7+\frac{\alpha _w \Gamma _1^*}{|\textsf{u}|_1}{\mathcal X}_2\right) {\mathcal X}_3+k_8{\mathcal X}^*_9+k_{10}e_S\left( \alpha _{10}+\frac{\alpha _w \Gamma _1^*}{|\textsf{u}|_1}{\mathcal X}_1\right) {\mathcal X}_2+k_{11}{\mathcal X}^*_{11}\nonumber \\ &  \hspace{1.0cm}=k_7\left( \left( \frac{2k_8+k_9}{k_8+k_9}\right) \alpha _7+\frac{\alpha _w}{|\textsf{u}|_1}\left( \Gamma _1^*+\frac{k_8}{k_8+k_9}\Phi _1^*\right) {\mathcal X}_2\right) e_H{\mathcal X}_3+\end{aligned}$$

D19 $$\begin{aligned} &  \hspace{1.5cm} k_{10}\left( \left( \frac{2k_{11}+k_{12}}{k_{11}+k_{12}}\right) \alpha _{10}+\frac{\alpha _w}{|\textsf{u}|_1}\left( \Gamma _1^*+\frac{k_{11}}{k_{11}+k_{12}}\Phi _1^*\right) {\mathcal X}_1\right) e_S{\mathcal X}_2 \end{aligned}$$

respectively, where

$$\begin{aligned} {\mathcal X}^*_5=\frac{k_1\frac{e_0}{\sqrt{N_c}}|\textsf{u}|_1(\alpha _1+\frac{\alpha _w\Phi _1^*}{|\textsf{u}|_1}{\mathcal X}_1){\mathcal X}_3}{k_2+k_3+k_1|\textsf{u}|_1(\alpha _1+\frac{\alpha _w\Phi _1^*}{|\textsf{u}|_1}{\mathcal X}_1){\mathcal X}_3} \end{aligned}$$

$$\begin{aligned} {\mathcal X}^*_7=\frac{k_4\frac{u_0}{\sqrt{N_c}}|\textsf{u}|_1(\alpha _4+\frac{\alpha _w\Phi _1^*}{|\textsf{u}|_1}{\mathcal X}_2){\mathcal X}_1}{k_5+k_6+k_4|\textsf{u}|_1(\alpha _4+\frac{\alpha _w\Phi _1^*}{|\textsf{u}|_1}{\mathcal X}_2){\mathcal X}_1} \end{aligned}$$

$$\begin{aligned} {\mathcal X}^*_9=\frac{k_7\frac{h_0}{\sqrt{N_c}}|\textsf{u}|_1(\alpha _7+\frac{\alpha _w\Phi _1^*}{|\textsf{u}|_1}{\mathcal X}_2){\mathcal X}_3}{k_8+k_9+k_7|\textsf{u}|_1(\alpha _7+\frac{\alpha _w\Phi _1^*}{|\textsf{u}|_1}{\mathcal X}_2){\mathcal X}_3} \end{aligned}$$

$$\begin{aligned} {\mathcal X}^*_{11}=\frac{k_{10}\frac{s_0}{\sqrt{N_c}}|\textsf{u}|_1(\alpha _{10}+\frac{\alpha _w\Phi _1^*}{|\textsf{u}|_1}{\mathcal X}_1){\mathcal X}_2}{k_{11}+k_{12}+|\textsf{u}|_1\frac{B}{\sqrt{N_c}}+k_{10}|\textsf{u}|_1(\alpha _{10}+\frac{\alpha _w\Phi _1^*}{|\textsf{u}|_1}{\mathcal X}_1){\mathcal X}_2} \end{aligned}$$

$$\begin{aligned} e_2=\frac{\frac{e_0}{\sqrt{N_c}}(k_2+k_3)}{k_2+k_3+k_1|\textsf{u}|_1(\alpha _1+\frac{\alpha _w\Phi _1^*}{|\textsf{u}|_1}{\mathcal X}_1){\mathcal X}_3} \end{aligned}$$

$$\begin{aligned} e_U=\frac{\frac{u_0}{\sqrt{N_c}}(k_5+k_6)}{k_5+k_6+k_4|\textsf{u}|_1(\alpha _4+\frac{\alpha _w\Phi _1^*}{|\textsf{u}|_1}{\mathcal X}_2){\mathcal X}_1} \end{aligned}$$

$$\begin{aligned} e_H=\frac{\frac{h_0}{\sqrt{N_c}}(k_8+k_9)}{k_8+k_9+k_7|\textsf{u}|_1(\alpha _7+\frac{\alpha _w\Phi _1^*}{|\textsf{u}|_1}{\mathcal X}_2){\mathcal X}_3} \end{aligned}$$

$$\begin{aligned} e_S=\frac{\frac{s_0}{\sqrt{N_c}}(k_{11}+k_{12})}{k_{11}+k_{12}+|\textsf{u}|_1\frac{B}{\sqrt{N_c}}+k_{10}|\textsf{u}|_1(\alpha _{10}+\frac{\alpha _w\Phi _1^*}{|\textsf{u}|_1}{\mathcal X}_1){\mathcal X}_2} \end{aligned}$$
