# Supplementary material for: Characterising Epigenetic Tipping Points using a Spectral Dimension Reduction Approach
Source: Bull Math Biol. 2026 Mar 18;88(4):57. doi: 10.1007/s11538-026-01602-w (PMC12999790; doi:10.1007/s11538-026-01602-w)
Supplement: Supplementary file 1 — Supplementary information: A supplementary materials and methods file accompanies this manuscript, which also includes supplementary figures and results. (pdf 1,708KB) [file 11538_2026_1602_MOESM1_ESM.pdf]

Characterising epigenetic tipping points using a spectral dimension reduction approach

Supplementary Materials

Tomás Alarcón*
ICREA, Pg. Lluís Companys 23, 08010 Barcelona, Spain.
Centre de Recerca Matemàtica, Edifici C, Campus de Bellaterra, 08193 Bellaterra (Barcelona), Spain.
Departament de Matemàtiques, Universitat Autònoma de Barcelona, 08193 Bellaterra (Barcelona), Spain. and
Barcelona Collaboratorium for Theoretical & Predicitive Biology,
Pasqual Maragall Foundation Building, Wellington, 30, Barcelona, 08005, Spain

Javier A. Menendez
Program Against Cancer Therapeutic Resistance (ProCURE),
Metabolism and Cancer Group, Catalan Institute of Oncology, 17007 Girona, Spain. and
Girona Biomedical Research Institute, 17190 Salt, Girona, Spain.

Josep Sardanyés
Centre de Recerca Matemàtica, Edifici C, Campus de Bellaterra, 08193 Bellaterra (Barcelona), Spain.

I. SUPPLEMENTARY METHODS: CONNECTIVITY MATRIX

We consider three types of interactions and their associated matrices, namely, all-to-all interactions, "Hi-C-like" interactions, and first-neighbor interactions. This choice was guided by previous work on this topic, as most previous models of bistable epigenetic regulation assume all-to-all interactions [1–3], sometimes considering a kernel so that the probability of connection between two sites decreases with the distance between them. The matrix associated with this interaction pattern is generated as follows. The off-diagonal elements of the matrix were sampled from a uniform distribution, i.e. $w_{ij} = w_0 U_{ij}$, where $U_{ij} \sim \mathrm{U}(0, 1)$ are random numbers uniformly distributed between 0 and 1. The quantity w_0 is a global scale for the intensity of the interactions between sites. The diagonal elements are also randomly generated and given by $w_{ii} = \omega_0 w_0 U_{ii}$, where $\omega_0 > 1$ is introduced to reproduce the feature usually observed in data that the self-interactions are stronger than the interactions with other sites (in this case we have chosen $\omega_0 = 5$). Finally, we symmetrize the matrix. In this way, we obtained a pattern of interactions where, on average, the intensity of the interaction between any pair of sites is the same.

The matrix corresponding to first-neighbor interactions has been generated as follows. First, we have generated a band diagonal matrix with $w_{ii} = \omega_0 w_0 U_{ii}$, $w_{i,i+1} = w_0 U_{i,i+1}$, and $w_{i-1,i} = w_0 U_{i,i-1}$, where U_{ii}, $U_{i,i+1}$, and $U_{i,i-1}$ are random numbers uniformly distributed between 0 and 1. Since, as per the Perron-Frobenius theorem, the spectral reduction method requires that all the matrix elements be strictly positive, i.e. $w_{ij} > 0$, we have set the elements off the band to $w_{ij} = \epsilon_0 U_{ij}$ with $\epsilon_0 \ll 1$ (specifically, we have set $\epsilon_0 = 10^{-2}$). As in the previous case, we finalize by symmetrizing the matrix.

Finally, we have considered a third pattern of interaction that reproduces the qualitative features observed in Hi-C interaction maps. At the scales considered in this paper, the map of interactions is heterogeneous (see [4, 5]). It exhibits sub-regions that are tightly connected, whereas the connections between such sub-regions are much weaker or infrequent. It can thus be considered an intermediate case between the first-neighbor and the all-to-all case: the general level of connectivity is much larger than in the first-neighbor pattern but not so dense as in the all-to-all connectivity. To analyze this case in the context of our coarse-graining methodology, we have generated a matrix that has the same qualitative features as the matrix obtained from Hi-C data. Specifically, our matrix emulates the data provided in [4], Fig. 2. The matrices used in our analysis are shown in Fig. 1.

The ablation study shown in Fig. 4(d) of the main text involves taking $w_{ij} = 0$, for all $i \neq j$. This is an issue regarding the spectral dimension reduction method, as it assumes that the matrix W is such that W^T has a dominant eigenvalue $\lambda_D > 0$ with an associated dominant eigenvector $\mathsf{u} = (u_i)_{i=1}^{N_S}$ such that $u_i > 0$ for all i. The conditions under which this is true are enshrined in the Perron-Frobenius theorem, which requires $w_{ij} > 0$ for all i and j. Therefore, our CG methodology cannot be applied to the ablation study as suggested.

* Corrsponding author: talarcon@crm.cat

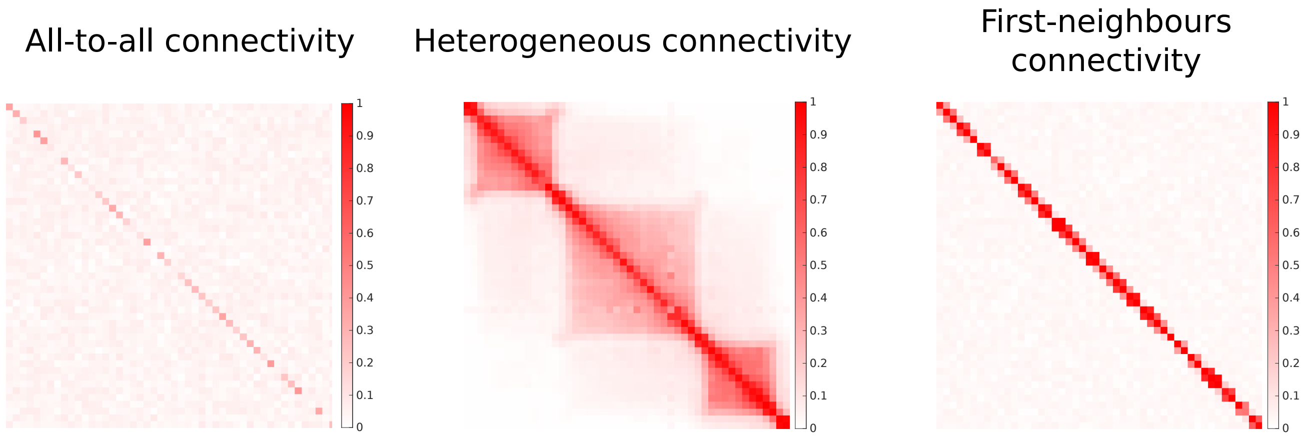

FIG. 1. **Connectivity patterns.** Graphical representation of the matrices considered in our analysis corresponding to the three patterns of interactions between bins described in the text, namely, all-to-all, heterogeneous, and first-neighbor. For the purpose of this graphical representation, the entries of the matrix have been normalized to be between zero and one.

However, although we cannot perform the CG reduction in the ablation study, the microscopic model exhibits no such restrictions, as we can make direct numerical simulations of the microscopic dynamics (however costly). We have done it with a first-neighbor interaction matrix (defined as explained in our reply to Comment 1, by setting $\epsilon_0 = 0$). We will refer to this matrix as the "ablated first-neighbor" (AFN) matrix. The results of this analysis are shown in Fig. YYY, which shows simulations obtained by numerically solving the system of ordinary differential equations of the microscopic model with the AFN matrix for $N_S = 48$ i.e., 48 genomic bins. Specifically, for different values of the DNA damage, B, we have let the system reach its steady state, i.e., $(m_i^*, a_i^*)_{i=1}^{N_S}$. We have then plotted the steady-state value of the average methylation and average acetylation, given by,

$$\bar{M} = \frac{1}{N_S}\sum_{i=1}^{N_S} m_i^* \; \bar{A} = \frac{1}{N_S}\sum_{i=1}^{N_S} a_i^*,$$

respectively, as we vary B, as shown in Figs. 4(c.2) and (c.3) of the main text.

II. SUPPLEMENTARY METHODS: WKB ANALYSIS

Asymptotic solution of the averaged FP equation for the slow variables

At this point we can calculate the analytic expression of the steady state PDF, $\psi_s(\mathcal{X}_s)$, by resorting to the Wentzel–Kramers–Brillouin (WKB) approximation [6–9], which in dimensions higher than 1 is sometimes referred to as "ray theory" [10], of the steady state averaged FP equation, Eq. (**??**):

$$\langle \mathcal{L}_s \rangle [\psi_s(\mathcal{X}_s)] = 0.$$

The main tenet of the WKB approximation is the so-called *WKB Ansatz*, i.e., that the solution to this equation can be approximated (when $\Omega_{27} \gg 1$) by a function of the form:

$$\phi_s(\mathcal{X}_s) \simeq \mathcal{R}(\mathcal{X}_s) e^{\Omega_{27}\mathcal{S}(\mathcal{X}_s)}.$$

By introducing the WKB Ansatz into the steady-state averaged FP, Eq. (**??**), and solving order-to-order in powers of Ω_{27}, we obtain:

Leading order ($\mathcal{O}(\Omega_{27})$). At leading order, one can easily show that $\mathcal{S}$ satisfies a Hamilton-Jacobi equation (HME):

$$\langle \mathcal{H} \rangle (\mathcal{X}_s, \partial_s \mathcal{S}) = 0 \tag{1}$$

where the averaged Hamiltonian, $\langle\mathcal{H}\rangle$, is given by:

$$\langle\mathcal{H}\rangle(\mathcal{X}_s,\mathcal{P}_s)=\sum_{i=1,2}\left(-\langle\mathcal{D}_i\rangle\mathcal{P}_i+\frac{1}{2}\langle\mathcal{N}_i\rangle\mathcal{P}_i^2\right). \tag{2}$$

It is well known that the solution of Eq. (1) can be written as

$$\mathcal{S}(\mathcal{X}_s)=\int_{\mathcal{X}_0}^{\mathcal{X}_s}\mathcal{P}_s d\mathcal{X}_s \tag{3}$$

where $\mathcal{X}_s$ and $\mathcal{P}_s$ are the solutions of the Hamilton equations

$$\begin{aligned}\frac{d\mathcal{X}_i}{ds}&=-\partial_{\mathcal{P}_i}\langle\mathcal{H}\rangle=\langle\mathcal{D}_i\rangle-\langle\mathcal{N}_i\rangle\mathcal{P}_i\\ \frac{d\mathcal{P}_i}{ds}&=\partial_{\mathcal{X}_i}\langle\mathcal{H}\rangle=\sum_{i=1,2}\left(-(\partial_i\langle\mathcal{D}_i\rangle)\mathcal{P}_i+\frac{1}{2}(\partial_i\langle\mathcal{N}_i\rangle)\mathcal{P}_i^2\right)\end{aligned} \tag{4}$$

with "initial" conditions $\mathcal{X}_s(s=0)=\mathcal{X}_0$ and $\mathcal{P}_s(s=0)=\mathcal{P}_0$. One must take into account that, since $\langle\mathcal{H}\rangle=0$ is a conserved quantity, the initial conditions must satisfy $\langle\mathcal{H}\rangle(\mathcal{X}_0,\mathcal{P}_0)=0$.

The subleading order ($\mathcal{O}(\Omega_{27}^0)$) provides a transport equation for the lowest order approximation of the prefactor $\mathcal{R}$. Since the leading order approximation is enough for our analysis, we do not tackle the subleading approximation. The reader is referred to [9] for further details on this issue.

a. Detailed-balance solution. Further analytical progress can be achieved by imposing that detailed-balance holds so that

$$\mathcal{H}_i(\mathcal{X}_i,\mathcal{P}_i)=\langle\mathcal{D}_i\rangle\mathcal{P}_i-\frac{1}{2}\langle\mathcal{N}_i\rangle\mathcal{P}_i^2=0,\ i\in slow$$

which implies that

$$\mathcal{P}_i=0,\text{ or }\mathcal{P}_i=2\frac{\langle\mathcal{D}_i\rangle}{\langle\mathcal{N}_i\rangle}.$$

According to the definition of $\mathcal{S}_0$, we have that

$$\partial_{\mathcal{X}_i}\mathcal{S}_0=\mathcal{P}_i$$

so that

$$\partial_{\mathcal{X}_i}\mathcal{S}_0=0\Rightarrow\mathcal{P}_i=2\frac{\langle\mathcal{D}_i\rangle}{\langle\mathcal{N}_i\rangle}=0$$

and therefore $\mathcal{X}_s^*$ correspond to the fixed point(s) of the deterministic system.

Furthermore, under this assumption, the Hamilton equations simplify to a set of two ODEs:

$$\dot{\mathcal{X}}_i=-\langle\mathcal{D}_i\rangle,\ \mathcal{P}_i(\boldsymbol{\mathcal{X}}_s)=2\frac{\langle\mathcal{D}_i\rangle}{\langle\mathcal{N}_i\rangle} \tag{5}$$

Note that Eqs. (5) correspond to the time-reversal of the mean-field equations.

b. Computation of the $\nabla\left(\Delta\mathcal{S}_\pm(\Theta)\right)$*.* Within this approximation we can obtain an estimation of the gradient of $\Delta\mathcal{S}_\pm(\Theta)$ (see Eq. (2)) of the main paper. We have that, in general, according to the WKB approximation:

$$\Delta\mathcal{S}_\pm(\Theta)=\int_{\boldsymbol{\mathcal{X}}_0}^{\boldsymbol{\mathcal{X}}_\pm}\boldsymbol{\mathcal{P}}\cdot d\boldsymbol{\mathcal{X}}=\int_{t_0}^{t_f}\left(\mathcal{P}\cdot\dot{\mathcal{X}}\right)dt$$

where the integral is calculated on the trajectory corresponding to the solution of Eqs. (5), so that, by making explicit the dependence on $\Theta=\{\theta_i\}_{i=1,\dots,D_p}$

$$\Delta\mathcal{S}_{\pm}(\Theta) = -\int_{t_0}^{t_f} \left(\mathcal{P}(\boldsymbol{\mathcal{X}}_s(t),\Theta)\cdot\boldsymbol{\mathcal{D}}(\boldsymbol{\mathcal{X}}_s(t),\Theta)\right) dt$$

so that

$$\partial_{\theta_i}\Delta\mathcal{S}_{\pm}(\Theta) = -\int_{t_0}^{t_f} \frac{\partial}{\partial\theta_i}\left(\mathcal{P}(\boldsymbol{\mathcal{X}}_s(t),\Theta)\cdot\boldsymbol{\mathcal{D}}(\boldsymbol{\mathcal{X}}_s(t),\Theta)\right) dt.$$

III. SUPPLEMENTARY METHODS: ANALYSIS OF THE COARSE-GRAINED MODEL WITH ACETYL-COA COFACTOR

HM and HAC enzymes need a cofactor that acts as a donor of the methyl and acetyl groups, respectively. The most common of such cofactors are S-Adenosyl methionine (SAM), a methyl donor for many HMs including EZH2, and the actyl donor Acetyl-coA. In this section, we discuss how to include these cofactors in the model. The following discussion focuses on the HAC-Acetyl-coA model. The model corresponding to the regulation of HM activity by SAM is completely analogous.

a. Model with HAC cofactor. Our sensitivity analysis (see Main Text) has revealed that the parameters that (up)regulate the activity of the histone acetylase (HAC) are the ones (together with the concentrations of chromatin-modifying enzymes) that have the biggest effect on the robustness of the hypoacetylated state. In order to investigate this further, regarding the identification of early-warning signals associated with the loss of the hypoacetylated state, we invoke the fact that the chromatin modifiers considered in this work, specifically, the HAC, depend on the abundance of metabolic cofactors [11]. Cofactors are a well-known regulatory mechanism of enzymatic activity. In a nutshell, the regulated enzyme has no activity unless the cofactor activates it. Such activation occurs, for example, by forming a complex with the enzyme that has the capacity of transforming the substrate into the product [11]. HACs have acetyl-coA (A-coA) as a cofactor that acts as the donor of the acetyl group that will be attached to the H3K27 residue. According to the aforementioned model, the acetylation reaction must be modified to account for the intermediate step of forming a HAC-A-coA complex, which then goes on to carry out the enzymatic transformation of H3K27 (non-acetylated) into H3K27ac (acetylated):

$$E_H + A \underset{k_{16}}{\overset{k_{15}}{\rightleftharpoons}} E_{HA}$$

$$S_i + E_{HA} \underset{k_{8_i}}{\overset{k_{7_i}}{\rightleftharpoons}} C_{HA_i} \xrightarrow{k_{9_i}} A_i + E_{HA}.$$

Here, E_H: number of free HAC, A: number of free acetyl-coA, E_{HA}: number of activated HAC, C_{HA_i}: number of H3K27-activated HAC complexes, A_i: number of acetylated residues (H3K27ac). The conservation law associated with the HAC now reads:

$$E_H + E_{HA} + \sum_i C_{HA_i} = H_0.$$

The stochastic model including HAC-regulation by acetyl-coA is described in detail in Table II. For simplicity, we will consider that (i) A is constant (i.e., we consider there is a reservoir of this substance provided by the cell's metabolism), and (ii) the formation of the complex (activated HAC) E_{HA} is fast and therefore in quasi-equilibrium with the other reactions (the same assumptions that we consider regarding the sequestration of SIRT by DNA damaged sites).

Coarse-grained model

The reaction rates corresponding to the model with acetyl-coA as a cofactor regulating the activity of the histone acetylase (HAC) enzyme are given in Table II. Following the procedure outlined above, we can derive the corresponding coarse-grained equations for the observables. Similarly to our assumption regarding the sequestration of HDAC by non-chromatin targets (e.g., DNA damaged sites), we will consider that (i) the formation of the HDAC-acetyl-coA,

denoted as e_{HA}, is very fast (i.e., in quasiequilibrium with the rest of the system), and (ii) the system is assumed to be in a "bath" of acetyl-coA, so that its concentration, $\mathcal{A}_T$, can be considered to be constant. Under these assumptions, the coarse-grained model reads as follows.

For the fast variables:

1. EZH2-unmodified chromatin complex ($\mathcal{C}_2$), $\mathcal{X}_5$
   - $\mathcal{D}_5 = k_1\alpha_1 e_{2S}\mathcal{X}_3 - (k_2+k_3)\mathcal{X}_5 + \frac{k_1\alpha_w\Phi_1^*}{|\mathsf{u}|_1}e_{2S}\mathcal{X}_1\mathcal{X}_3.$
   - $\mathcal{N}_5 = k_1\alpha_1 e_{2S}\mathcal{X}_3 + (k_2+k_3)\mathcal{X}_5 + \frac{k_1\alpha_w\Gamma_1^*}{|\mathsf{u}|_1}e_{2S}\mathcal{X}_1\mathcal{X}_3.$
2. UTX-methylated chromatin complex ($\mathcal{C}_U$), $\mathcal{X}_7$
   - $\mathcal{D}_7 = k_4\alpha_4 e_U\mathcal{X}_1 - (k_5+k_6)\mathcal{X}_7 + \frac{k_4\alpha_w\Phi_1^*}{|\mathsf{u}|_1}e_U\mathcal{X}_2\mathcal{X}_1.$
   - $\mathcal{N}_7 = k_4\alpha_4 e_U\mathcal{X}_1 + (k_5+k_6)\mathcal{X}_7 + \frac{k_4\alpha_w\Gamma_1^*}{|\mathsf{u}|_1}e_U\mathcal{X}_2\mathcal{X}_1.$
3. Histone acetylase-unmodified chromatin complex ($\mathcal{C}_H$), $\mathcal{X}_9$
   - $\mathcal{D}_9 = k_7\alpha_7 e_{HA}\mathcal{X}_3 - (k_8+k_9)\mathcal{X}_9 + \frac{k_7\alpha_w\Phi_1^*}{|\mathsf{u}|_1}e_{HA}\mathcal{X}_2\mathcal{X}_3.$
   - $\mathcal{N}_9 = k_7\alpha_7 e_{HA}\mathcal{X}_3 + (k_8+k_9)\mathcal{X}_9 + \frac{k_7\alpha_w\Gamma_1^*}{|\mathsf{u}|_1}e_{HA}\mathcal{X}_2\mathcal{X}_3.$
4. Histone deacetylase (sirtuin)-acetylated chromatin complex ($\mathcal{C}_S$), $\mathcal{X}_{11}$
   - $\mathcal{D}_{11} = k_{10}\alpha_{10} e_S\mathcal{X}_2 - (k_{11}+k_{12})\mathcal{X}_{11} + \frac{k_{10}\alpha_w\Phi_1^*}{|\mathsf{u}|_1}e_S\mathcal{X}_1\mathcal{X}_2.$
   - $\mathcal{N}_{11} = k_{10}\alpha_{10} e_S\mathcal{X}_2 + (k_{11}+k_{12})\mathcal{X}_{11} + \frac{k_{10}\alpha_w\Gamma_1^*}{|\mathsf{u}|_1}e_S\mathcal{X}_1\mathcal{X}_2.$

The (mean-field) quasi-steady state approximation is:

1. EZH2-unmodified chromatin complex

$$\mathcal{X}_5^* = \frac{k_1\frac{e_0}{\sqrt{N_c}}|\mathsf{u}|_1\hat{s}_c(\alpha_1+\frac{\alpha_w\Phi_1^*}{|\mathsf{u}|_1}\mathcal{X}_1)\mathcal{X}_3}{k_2+k_3+k_1|\mathsf{u}|_1\hat{s}_c(\alpha_1+\frac{\alpha_w\Phi_1^*}{|\mathsf{u}|_1}\mathcal{X}_1)\mathcal{X}_3}.$$

2. UTX-methylated chromatin complex

$$\mathcal{X}_7^* = \frac{k_4\frac{u_0}{\sqrt{N_c}}|\mathsf{u}|_1(\alpha_4+\frac{\alpha_w\Phi_1^*}{|\mathsf{u}|_1}\mathcal{X}_2)\mathcal{X}_1}{k_5+k_6+k_4|\mathsf{u}|_1(\alpha_4+\frac{\alpha_w\Phi_1^*}{|\mathsf{u}|_1}\mathcal{X}_2)\mathcal{X}_1}.$$

3. Histone acetylase-unmodified chromatin complex

$$\mathcal{X}_9^* = \frac{k_7\frac{h_0}{\sqrt{N_c}}|\mathsf{u}|_1\hat{a}_c(\alpha_7+\frac{\alpha_w\Phi_1^*}{|\mathsf{u}|_1}\mathcal{X}_2)\mathcal{X}_3}{k_8+k_9+k_7|\mathsf{u}|_1\hat{a}_c(\alpha_7+\frac{\alpha_w\Phi_1^*}{|\mathsf{u}|_1}\mathcal{X}_2)\mathcal{X}_3}.$$

4. Histone deacetylase (sirtuin)-acetylated chromatin complex

$$\mathcal{X}_{11}^* = \frac{k_{10}\frac{s_0}{\sqrt{N_c}}|\mathsf{u}|_1(\alpha_{10}+\frac{\alpha_w\Phi_1^*}{|\mathsf{u}|_1}\mathcal{X}_1)\mathcal{X}_2}{k_{11}+k_{12}+|\mathsf{u}|_1\frac{B}{\sqrt{N_c}}+k_{10}|\mathsf{u}|_1(\alpha_{10}+\frac{\alpha_w\Phi_1^*}{|\mathsf{u}|_1}\mathcal{X}_1)\mathcal{X}_2}.$$

where the quantities $\hat{a}_c$ and $\hat{s}_c$ are given by

$$\hat{a}_c = \frac{\frac{k_{15}}{k_{16}}\mathcal{A}_T}{\frac{|u|_1}{\sqrt{N_c}}\frac{k_{15}}{k_{16}}\mathcal{A}_T+1}$$

and

$$\hat{s}_c = \frac{\frac{k_{17}}{k_{18}}\mathcal{S}_T}{\frac{|u|_1}{\sqrt{N_c}}\frac{k_{17}}{k_{18}}\mathcal{S}_T + 1},$$

respectively. Note that, due to renormalization of the conservation laws in the coarse-graining procedure, the counterpart of the quantity $\hat{a}_c$ in the microscopic model, $\hat{a}_m$, is given by

$$\hat{a}_m = \frac{\frac{k_{15}}{k_{16}}\mathcal{A}_T}{\frac{k_{15}}{k_{16}}\mathcal{A}_T + 1}.$$

The microscopic analogous of $\hat{s}_c$ is

$$\hat{s}_m = \frac{\frac{k_{17}}{k_{18}}\mathcal{S}_T}{\frac{k_{17}}{k_{18}}\mathcal{S}_T + 1}.$$

Finally, the coarse-grained dynamics for the slow observables (weighted methylation and weighted acetylation) is given by:

1. Weighted methylation

$$\langle \mathcal{D}_1 \rangle = k_3\mathcal{X}_5^* - k_6\mathcal{X}_7^* \tag{6}$$

$$\begin{aligned}\langle \mathcal{N}_1 \rangle &= k_1 e_{2S}\left(\alpha_1 + \frac{\alpha_w\Gamma_1^*}{|\mathsf{u}|_1}\mathcal{X}_1\right)\mathcal{X}_3 + k_2\mathcal{X}_5^* + k_4 e_U\left(\alpha_4 + \frac{\alpha_w\Gamma_1^*}{|\mathsf{u}|_1}\mathcal{X}_2\right)\mathcal{X}_1 + k_5\mathcal{X}_7^* \\ &= k_1\left(\left(\frac{2k_2+k_3}{k_2+k_3}\right)\alpha_1 + \frac{\alpha_w}{|\mathsf{u}|_1}\left(\Gamma_1^* + \frac{k_2}{k_2+k_3}\Phi_1^*\right)\mathcal{X}_1\right)e_{2S}\mathcal{X}_3 + \\ &\quad k_4\left(\left(\frac{2k_5+k_6}{k_5+k_6}\right)\alpha_4 + \frac{\alpha_w}{|\mathsf{u}|_1}\left(\Gamma_1^* + \frac{k_5}{k_5+k_6}\Phi_1^*\right)\mathcal{X}_2\right)e_U\mathcal{X}_1\end{aligned} \tag{7}$$

2. Weighted acetylation

$$\langle \mathcal{D}_2 \rangle = k_9\mathcal{X}_9^* - k_{12}\mathcal{X}_{11}^* \tag{8}$$

$$\begin{aligned}\langle \mathcal{N}_2 \rangle &= k_7 e_{HA}\left(\alpha_7 + \frac{\alpha_w\Gamma_1^*}{|\mathsf{u}|_1}\mathcal{X}_2\right)\mathcal{X}_3 + k_8\mathcal{X}_9^* + k_{10} e_S\left(\alpha_{10} + \frac{\alpha_w\Gamma_1^*}{|\mathsf{u}|_1}\mathcal{X}_1\right)\mathcal{X}_2 + k_{11}\mathcal{X}_{11}^* \\ &= k_7\left(\left(\frac{2k_8+k_9}{k_8+k_9}\right)\alpha_7 + \frac{\alpha_w}{|\mathsf{u}|_1}\left(\Gamma_1^* + \frac{k_8}{k_8+k_9}\Phi_1^*\right)\mathcal{X}_2\right)e_{HA}\mathcal{X}_3 + \\ &\quad k_{10}\left(\left(\frac{2k_{11}+k_{12}}{k_{11}+k_{12}}\right)\alpha_{10} + \frac{\alpha_w}{|\mathsf{u}|_1}\left(\Gamma_1^* + \frac{k_{11}}{k_{11}+k_{12}}\Phi_1^*\right)\mathcal{X}_1\right)e_S\mathcal{X}_2\end{aligned} \tag{9}$$

where

1. Free EZH2

$$e_{2S} = \frac{\frac{e_0}{\sqrt{N_c}}(k_2+k_3)}{k_2 + k_3 + k_1|\mathsf{u}|_1\hat{s}_c(\alpha_1 + \frac{\alpha_w\Phi_1^*}{|\mathsf{u}|_1}\mathcal{X}_1)\mathcal{X}_3}$$

2. Free UTX

$$e_U = \frac{\frac{u_0}{\sqrt{N_c}}(k_5+k_6)}{k_5 + k_6 + k_4|\mathsf{u}|_1(\alpha_4 + \frac{\alpha_w\Phi_1^*}{|\mathsf{u}|_1}\mathcal{X}_2)\mathcal{X}_1}$$

3. Free HAC-Acetyl-coA

$$e_{HA} = \frac{\frac{h_0}{\sqrt{N_c}}(k_8+k_9)}{k_8 + k_9 + k_7|\mathsf{u}|_1\hat{a}_c(\alpha_7 + \frac{\alpha_w\Phi_1^*}{|\mathsf{u}|_1}\mathcal{X}_2)\mathcal{X}_3}$$

TABLE I. Table showing the reaction rates associated with the model of chromatin modifications. All the entries $r_{ijk} = 0$ except for those shown in the table below. $i = 1, \ldots, N_s$, $j = 1, \ldots, N_C$, and $k = 1, \ldots, R$. The number of species, regions, and reactions are denoted by $N_s = 12$, N_c, and $R = 14$, respectively.

Reaction	LAM rate	Non-zero stoichiometric coefficients		
$W_{(j-1)R+1}$	$k_{1_j} S_j E_2$	$r_{1j1} = -1$	$r_{4j1} = -1$	$r_{5j1} = +1$
$W_{(j-1)R+2}$	$k_{2_j} C_{E_j}$	$r_{1j2} = +1$	$r_{4j2} = +1$	$r_{5j2} = -1$
$W_{(j-1)R+3}$	$k_{3_j} C_{E_j}$	$r_{2j3} = +1$	$r_{5j3} = -1$	$r_{2j3} = +1$
$W_{(j-1)R+4}$	$k_{4_j} M_j E_U$	$r_{2j4} = -1$	$r_{6j4} = -1$	$r_{7j4} = +1$
$W_{(j-1)R+5}$	$k_{5_j} C_{U_j}$	$r_{2j5} = +1$	$r_{6j5} = +1$	$r_{7j7} = -1$
$W_{(j-1)R+6}$	$k_{6_j} C_{U_j}$	$r_{6j6} = +1$	$r_{7j6} = -1$	$r_{1j6} = +1$
$W_{(j-1)R+7}$	$k_{7_j} S_j E_H$	$r_{1j7} = -1$	$r_{8j7} = -1$	$r_{9j7} = +1$
$W_{(j-1)R+8}$	$k_{8_j} C_{H_j}$	$r_{1j8} = +1$	$r_{8j8} = +1$	$r_{9j8} = -1$
$W_{(j-1)R+9}$	$k_{9_j} C_{H_j}$	$r_{8j9} = +1$	$r_{9j9} = -1$	$r_{3j9} = +1$
$W_{(j-1)R+10}$	$k_{10_j} A_j E_S$	$r_{3j10} = -1$	$r_{10j10} = -1$	$r_{11j10} = +1$
$W_{(j-1)R+11}$	$k_{11_j} C_{S_j}$	$r_{3j11} = +1$	$r_{10j11} = +1$	$r_{11j11} = -1$
$W_{(j-1)R+12}$	$k_{12_j} C_{S_j}$	$r_{10j12} = +1$	$r_{11j12} = -1$	$r_{1j12} = +1$
$W_{(j-1)R+13}$	$k_{13_j} D E_S$	$r_{10j13} = -1$	$r_{12j13} = +1$	
$W_{(j-1)R+14}$	$k_{14_j} C_{S_D}$	$r_{10j14} = +1$	$r_{12j14} = -1$	

4. Free HAC

$$e_S = \frac{\frac{s_0}{\sqrt{N_c}}(k_{11} + k_{12})}{k_{11} + k_{12} + |\mathsf{u}|_1 \frac{B}{\sqrt{N_c}} + k_{10}|\mathsf{u}|_1(\alpha_{10} + \frac{\alpha_w \Phi_1^*}{|\mathsf{u}|_1}\mathcal{X}_1)\mathcal{X}_2}$$

Simulations assessing the performance of the coarse-graining procedure (mean-field case) are presented in Fig. 6.

b. Mean-field microscopic model with cofactors For clarity, we include here the mean-field equations for the microscopic model with cofactors.

$$\frac{dm_i}{dt} = k_3 \frac{k_1 e_0 \hat{s}_c \left(\alpha_1 + \sum_j w_{ij} m_j\right) s_i}{k_2 + k_3 + k_1 \left(\alpha_1 + \sum_j w_{ij} x_j\right) s_i} - k_6 \frac{k_4 u_0 \left(\alpha_1 + \sum_j w_{ij} a_j\right) m_i}{k_5 + k_6 + k4 \left(\alpha_1 + \sum_j w_{ij} a_j\right) m_i} \tag{10}$$

$$\frac{da_i}{dt} = k_9 \frac{k_7 h_0 \hat{a}_c \left(\alpha_7 + \sum_j w_{ij} a_j\right) s_i}{k_8 + k_9 + k_7 \left(\alpha_7 + \sum_j w_{ij} a_j\right) s_i} - k_{12} \frac{k_{10} e s_0 \left(\alpha_{10} + \sum_j w_{ij} m_j\right) a_i}{k_{11} + k_{12} + B + k_{10} \left(\alpha_{10} + \sum_j w_{ij} m_j\right) a_i} \tag{11}$$

where $i = 1, \ldots, N$.

IV. PARAMETER VALUES

$N = 48$
$k_1 = 10$
$k_4 = 5$
$k_7 = 5$
$k_{10} = 10$

$\alpha_1 = 0.1$
$\alpha_7 = 0.1$
$\alpha_4 = 0.1$
$\alpha_{10} = 10.0$

$k_2 = 1.0,\ k_3 = 1.0$
$k_5 = 1.0,\ k_6 = 1.0$
$k_8 = 1.0,\ k_9 = 1.0$
$k_{11} = 1.0,\ k_{12} = 1.0$

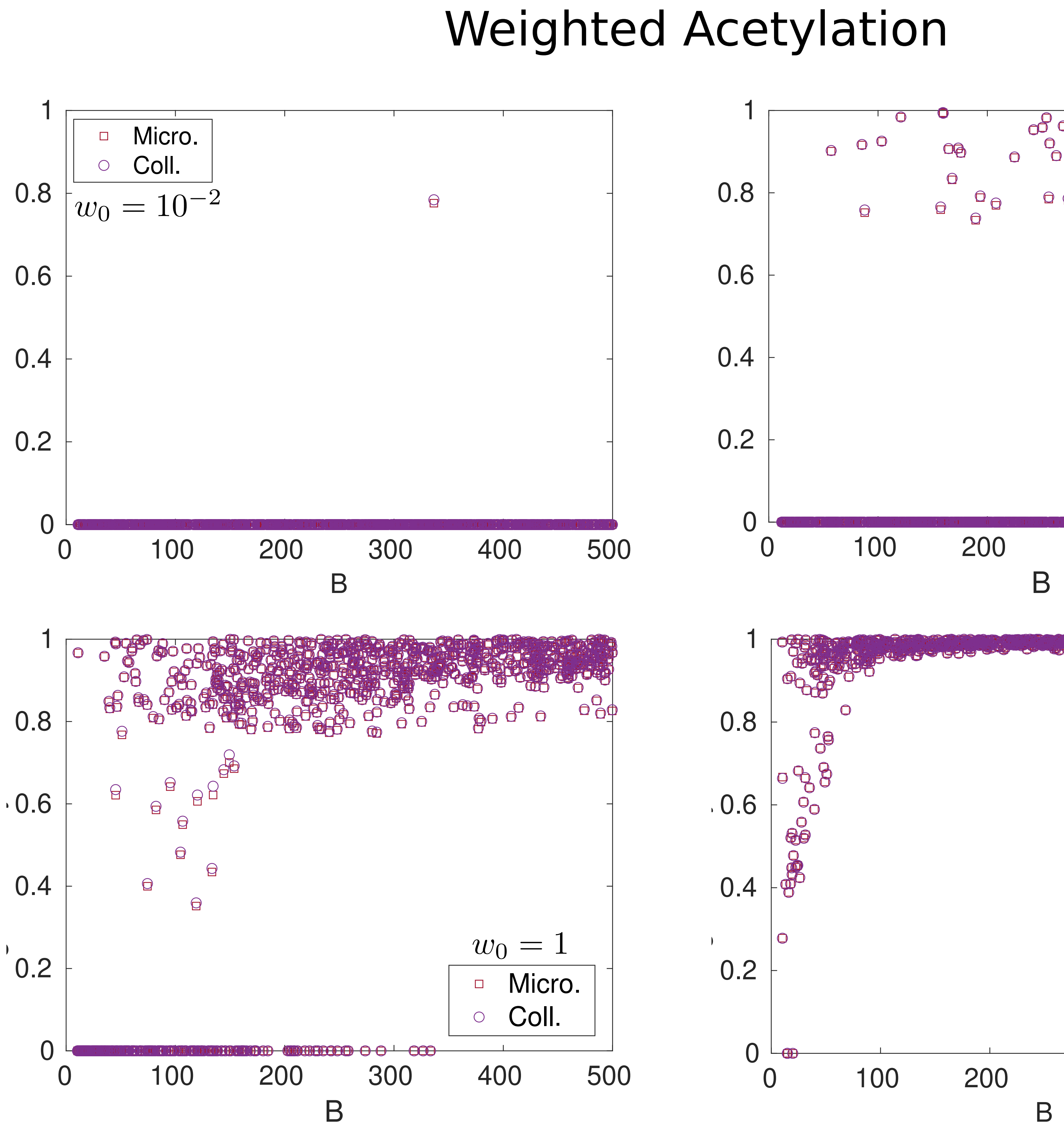

FIG. 2. **Benchmark simulations: Deterministic case.** These simulations show the comparison between the deterministic versions of the microscopic and the CG reduction for different values of the number of bins, N_C.

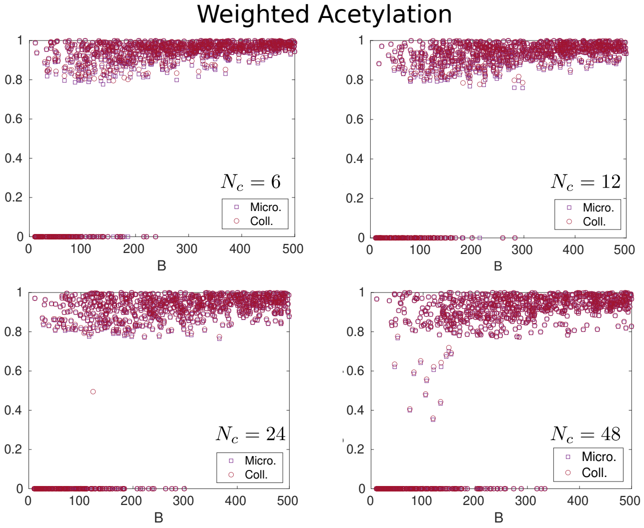

FIG. 3. **Benchmark simulations: Deterministic case.** Our model predicts that when the intensity of the connections between bins is either too strong or too weak, the ability of our system to exhibit bistable behavior is compromised. We show results in this figure comparing the microscopic with CG models with all-to-all connectivity for increasing values of the overall intensity of the interactions, w_0.

TABLE II. Table showing the reaction rates associated with the model of chromatin modifications with S-Adenosyl methionine and acetyl-coA cofactors. All the entries $r_{ijk} = 0$ except for those shown in the table below. $i = 1, \dots, N_s$, $j = 1, \dots, N_C$, and $k = 1, \dots, R$. The number of species, regions, and reactions are denoted by $N_s = 18$, N_c, and $R = 16$, respectively.

Reaction	LAM rate	Non-zero stoichiometric coefficients		
$W_{(j-1)R+1}$	$k_{1_j} S_j E_2$	$r_{1j1} = -1$	$r_{4j1} = -1$	$r_{5j1} = +1$
$W_{(j-1)R+2}$	$k_{2_j} C_{E_j}$	$r_{1j2} = +1$	$r_{4j2} = +1$	$r_{5j2} = -1$
$W_{(j-1)R+3}$	$k_{3_j} C_{E_j}$	$r_{2j3} = +1$	$r_{5j3} = -1$	$r_{2j3} = +1$
$W_{(j-1)R+4}$	$k_{4_j} M_j E_U$	$r_{2j4} = -1$	$r_{6j4} = -1$	$r_{7j4} = +1$
$W_{(j-1)R+5}$	$k_{5_j} C_{U_j}$	$r_{2j5} = +1$	$r_{6j5} = +1$	$r_{7j7} = -1$
$W_{(j-1)R+6}$	$k_{6_j} C_{U_j}$	$r_{6j6} = +1$	$r_{7j6} = -1$	$r_{1j6} = +1$
$W_{(j-1)R+7}$	$k_{7_j} S_j E_{HA}$	$r_{1j7} = -1$	$r_{8j7} = -1$	$r_{9j7} = +1$
$W_{(j-1)R+8}$	$k_{8_j} C_{HA_j}$	$r_{1j8} = +1$	$r_{8j8} = +1$	$r_{9j8} = -1$
$W_{(j-1)R+9}$	$k_{9_j} C_{HA_j}$	$r_{8j9} = +1$	$r_{9j9} = -1$	$r_{3j9} = +1$
$W_{(j-1)R+10}$	$k_{10_j} A_j E_S$	$r_{3j10} = -1$	$r_{10j10} = -1$	$r_{11j10} = +1$
$W_{(j-1)R+11}$	$k_{11_j} C_{S_j}$	$r_{3j11} = +1$	$r_{10j11} = +1$	$r_{11j11} = -1$
$W_{(j-1)R+12}$	$k_{12_j} C_{S_j}$	$r_{10j12} = +1$	$r_{11j12} = -1$	$r_{1j12} = +1$
$W_{(j-1)R+13}$	$k_{13_j} D E_S$	$r_{10j13} = -1$	$r_{12j13} = +1$	
$W_{(j-1)R+14}$	$k_{14_j} C_{S_D}$	$r_{10j14} = +1$	$r_{12j14} = -1$	
$W_{(j-1)R+15}$	$k_{15_j} A E_H$	$r_{13j15} = -1$	$r_{8j15} = +1$	
$W_{(j-1)R+16}$	$k_{16_j} E_{HA}$	$r_{13j16} = +1$	$r_{8j16} = -1$	
$W_{(j-1)R+17}$	$k_{15_j} S E_2$	$r_{13j15} = -1$	$r_{8j17} = +1$	
$W_{(j-1)R+18}$	$k_{16_j} E_{2S}$	$r_{13j16} = +1$	$r_{8j18} = -1$	

$k_{15} = 1.0, \; k_{16} = 1.0$
$k_{17} = 1.0, \; k_{18} = 1.0$

$e_0 = 1.0$
$u_0 = 1.0$
$h_0 = 1.0$
$es_0 = 1.0$

$s_0 = 1$

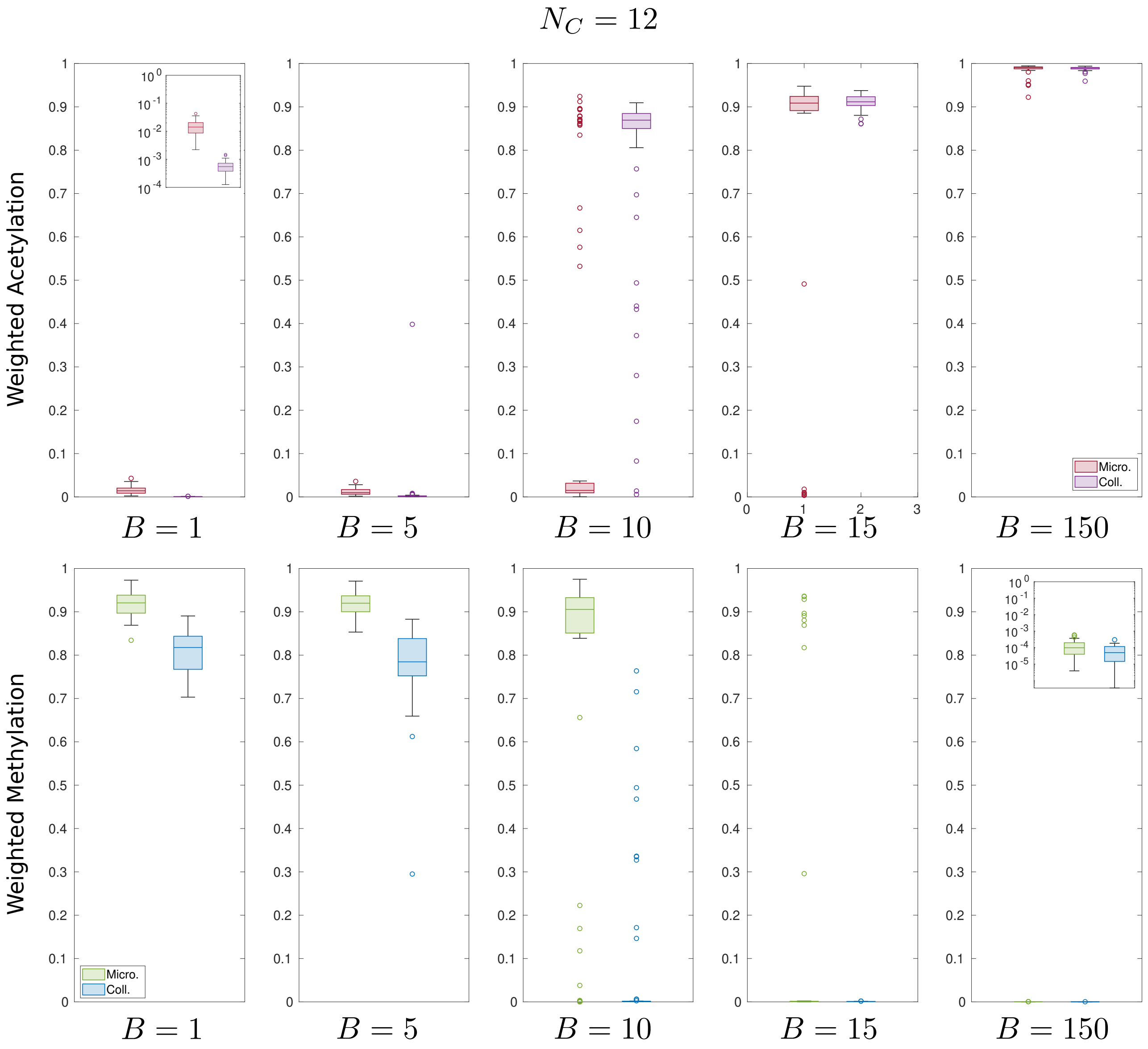

FIG. 4. **Benchmark simulations: Stochastic case.** Simulations illustrating the change of behavior in the system as we vary the DNA damage parameter, B. We compare the behavior of the diffusion limit for both the stochastic dynamics of the microscopic system and its CG reduction. The corresponding set of stochastic differential equations have been solved using the Ito-Euler method.

[1] I. B. Dodd, M. A. Micheelsen, K. Sneppen, and G. Thon, Theoretical analysis of epigenetic cell memory by nucleosome modification, Cell **129**, 813 (2007).

[2] K. Sneppen and L. Ringrose, Theoretical analysis of polycomb-trithorax systems predicts that poised chromatin is bistable and not bivalent, Nature Communications **10**, 2133 (2019).

[3] J. F. Nickels and K. Sneppen, Confinement mechanisms for epigenetic modifications of nucleosomes, PRX Life **1**, 013013 (2023).

[4] Q. Szabo, F. Bantignies, and G. Cavalli, Principles of genome folding into topologically associating domains, Science Advances **5**, 10.1126/sciadv.aaw1668 (2019).

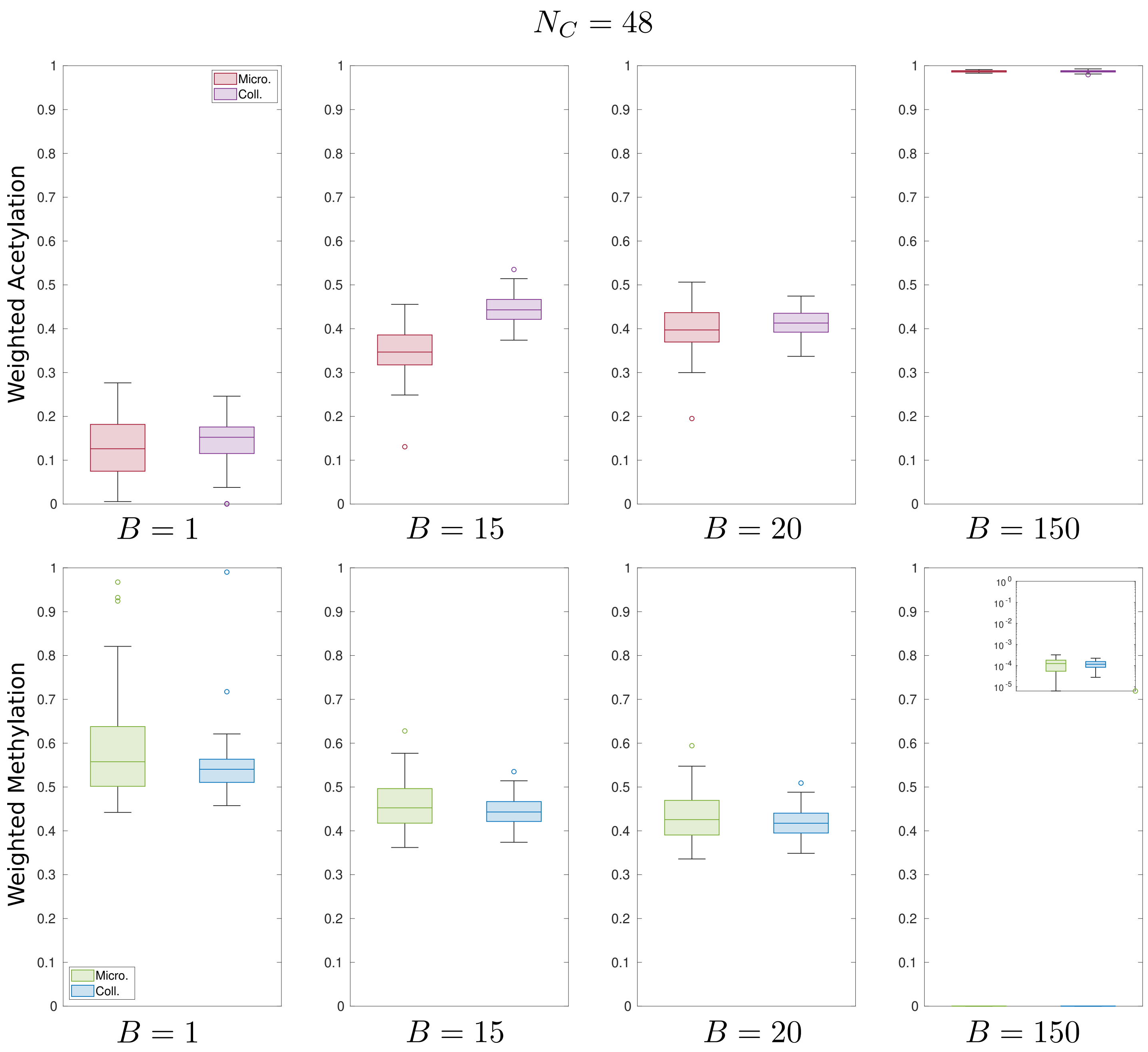

FIG. 5. **Benchmark simulations: Stochastic case.** Simulations illustrating the change of behavior in the system as we vary the DNA damage parameter, B. We compare the behavior of the diffusion limit for both the stochastic dynamics of the microscopic system and its CG reduction. The corresponding set of stochastic differential equations have been solved using the Ito-Euler method.

[5] H. A. Lawson, Y. Liang, and T. Wang, Transposable elements in mammalian chromatin, Nature Reviews Genetics **24**, 712 (2023).

[6] V. P. Maslov and M. V. Fedoriuk, *Semi-Classical Approximation in Quantum Mechanics* (Springer, Dordrecht, 1981).

[7] R. Hinch and S. J. Chapman, Exponentially slow transitions on a Markov chain: the frequency of calcium sparks, Eur. J. Appl. Math. **16**, 427 (2005).

[8] P. C. Bressloff, *Stochastic processes in cell biology.* (Springer-Verlag, Berlin, Germany, 2014).

[9] T. Bonnemain and D. Ullmo, Mean field games in the weak noise limit : A wkb approach to the fokker-planck equation, Physica A: Statistical Mechanics and its Applications **523**, 310 (2019).

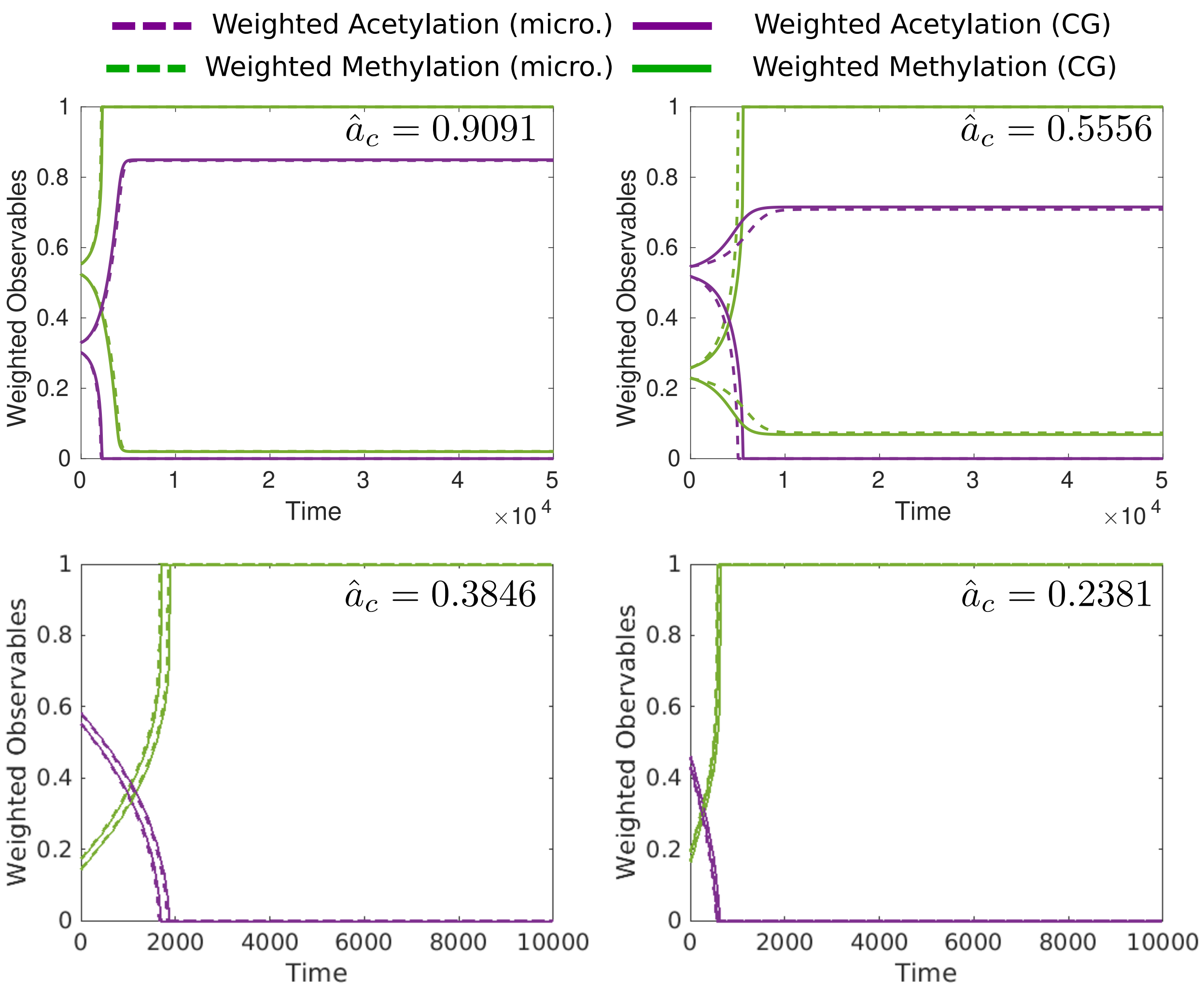

FIG. 6. **Benchmark simulations: Deterministic case.** Comparison between the microscopic and the coarse-grained dynamics for the system accounting for the finite abundance of the HAC cofactor Acetyl-coA. $B = 150$. $\hat{s}_c = 1$ in all simulations.

[10] J. K. Cohen and R. M. Lewis, A Ray Method for the Asymptotic Solution of the Diffusion Equation, IMA Journal of Applied Mathematics **3**, 266 (1967).

[11] B. P. Ingalls, *Mathematical modelling in systems biology. An introduction* (The MIT Press, Cambridge, Massachusets, USA, 2013).
